# Genome-wide association studies for yield-related traits in soft red winter wheat grown in Virginia

**DOI:** 10.1371/journal.pone.0208217

**Published:** 2019-02-22

**Authors:** Brian P. Ward, Gina Brown-Guedira, Frederic L. Kolb, David A. Van Sanford, Priyanka Tyagi, Clay H. Sneller, Carl A. Griffey

**Affiliations:** 1 Department Of Crop and Soil Environmental Sciences, Virginia Tech, Blacksburg, Virginia, United States of America; 2 Eastern Regional Small Grains Genotyping Laboratory, USDA-ARS, Raleigh, North Carolina, United States of America; 3 Department of Crop Sciences, University of Illinois, Urbana, Illinois, United States of America; 4 Department of Plant and Soil Sciences, University of Kentucky, Lexington, Kentucky, United States of America; 5 Department of Crop and Soil Sciences, North Carolina State University, Raleigh, North Carolina, United States of America; 6 Ohio Agricultural Research and Development Center, The Ohio State University, Wooster, Ohio, United States of America; USDA, UNITED STATES

## Abstract

Grain yield is a trait of paramount importance in the breeding of all cereals. In wheat (*Triticum aestivum* L.), yield has steadily increased since the Green Revolution, though the current rate of increase is not forecasted to keep pace with demand due to growing world population and increasing affluence. While several genome-wide association studies (GWAS) on yield and related component traits have been performed in wheat, the previous lack of a reference genome has made comparisons between studies difficult. In this study, a GWAS for yield and yield-related traits was carried out on a population of 322 soft red winter wheat lines across a total of four rain-fed environments in the state of Virginia using single-nucleotide polymorphism (SNP) marker data generated by a genotyping-by-sequencing (GBS) protocol. Two separate mixed linear models were used to identify significant marker-trait associations (MTAs). The first was a single-locus model utilizing a leave-one-chromosome-out approach to estimating kinship. The second was a sub-setting kinship estimation multi-locus method (FarmCPU). The single-locus model identified nine significant MTAs for various yield-related traits, while the FarmCPU model identified 74 significant MTAs. The availability of the wheat reference genome allowed for the description of MTAs in terms of both genetic and physical positions, and enabled more extensive post-GWAS characterization of significant MTAs. The results indicate a number of promising candidate genes contributing to grain yield, including an ortholog of the rice aberrant panicle organization (*APO1*) protein and a gibberellin oxidase protein (*GA2ox-A1*) affecting the trait grains per square meter, an ortholog of the *Arabidopsis thaliana* mother of flowering time and terminal flowering 1 (*MFT*) gene affecting the trait seeds per square meter, and a B2 heat stress response protein affecting the trait seeds per head.

## Introduction

Worldwide, wheat has the fourth-highest production of all crops, with a net production value that is second-highest of any crop [1]. In addition, wheat maintains the highest global harvested acreage of any crop [2]. To keep pace with an increasing world population and changes in diets due to increasing affluence, worldwide cereal production will have to increase by an estimated 50% over the period ending in 2050, requiring continuing genetic gains in yield potential of approximately 1.1% per year [3]. Sharma, et al. [4] estimated a historical average increase in grain yields in spring wheat of 0.65% per year when analyzing data across 15 years and 919 environments, while a recent study involving winter wheat in the Eastern United States estimated yearly increases in grain yield between 0.56% and 1.41%, depending upon environment [5].

Improvements to yield via direct selection are hampered by its highly quantitative and polygenic nature. Selection based on different yield component traits and crop physiology theory may offer additional avenues for increasing genetic gain in yield while avoiding yield plateaus [6]. However, relationships between traits must be taken into account, as there are many negative correlations between related traits, such as the well-documented negative correlation between the number of grains m^-2^ and average grain size [7]. A wide body of literature suggests that wheat is primarily sink-limited with respect to production of photosynthetic assimilates (reviewed in [8]). Supporting this theory, several studies have suggested that maximizing the number of seeds per unit area is critical for avoiding assimilate sink limitations and maximizing yield (reviewed in [9]). The two possible routes for increasing seeds per unit area are to: 1) increase the average number of seeds per head, and/or 2) increase the number of heads per unit area. Increases in yield will require both the production of greater numbers of grains, and an increase in the availability of photosynthates to prevent a corresponding drop in kernel sizes due to the compensation between these two traits [10]. Several previous studies have focused on increasing photosynthate availability via alterations to canopy light interception. Reynolds et al. [9] note that the amount of light interception is already nearly maximized in many wheat cultivars during the period after canopy closure and prior to leaf senescence. This leaves traits increasing the duration of light interception, e.g. faster canopy establishment through increased early-season vigor or later senescence via the “stay-green” trait [11], as appealing avenues for increasing photosynthate production.

In winter wheat, several genes are known to exert major effects on traits involved with seed production per unit area and photosynthesis duration. The time required for plants to flower and ultimately reach maturation is affected by multiple homeologous copies of the *VRN* vernalization requirement genes and the *PPD* photoperiod response genes [12]. Multiple major-effect quantitative trait loci (QTLs) affecting grain weight have also been identified, including *TaSus2-2B* [13], the *TaGW2* homeologous genes [14], and cell wall invertase (*Cwi*) genes [15]. However, it is likely that many genes affecting yield-related traits have yet to be identified. Genome-wide association studies (GWAS) and linkage mapping are the two predominate methods employed in plant breeding for associating phenotypic variation with underlying genetic variation. GWAS offers higher resolution due to many more ancestral gene recombinations within the testing panel, as opposed to only one or a few meiotic recombinations in a linkage mapping population. However, allele frequency is a primary factor limiting power in GWAS studies; marker-trait associations (MTAs) will be difficult or impossible to detect if causal variants are rare within the testing population [16]. GWAS methods utilizing mixed linear models have become standard methodology for their ability to guard against false positives due to population structure or kinship among genotypes [17]. Subsequent mixed model implementations have utilized multiple methods to increase power to detect loci of smaller effect. The multi-locus mixed model fits loci of large effect as covariates, allowing for the detection of more marker trait associations with smaller effects [18]. Recent methods including FaST-LMM-Select [19] and SUPER [20] have been developed which increase power by first performing a preliminary GWAS to identify the SNPs which are highly associated with the trait of interest, and subsequently using only this subset of SNPs when estimating kinship among lines in a second GWAS. Finally, the programs FarmCPU [21] and BLINK [22] combine the advantages of multi-locus mixed models with subsetting kinship estimation.

Relatively few GWAS analyses for yield and yield-related traits have been conducted in wheat, and of these, fewer still have been conducted in winter wheat germplasm [23–30]. Association studies have been more common in spring wheat, though the majority of these have been candidate-gene studies, with genome-wide studies being more limited [31]. Finally, it has been more common to perform GWAS in crop species using assembled diversity panels, rather than elite germplasm in current use by breeding programs [32]. GWAS in elite germplasm is typically limited to the identification of smaller-effect MTAs, as MTAs of major effect will have likely already become fixed within the mapping population [33]. Nevertheless, GWAS using panels of elite germplasm remain useful due to their higher relevance to the process of cultivar development [32]. One limitation of previous wheat GWAS studies was the lack of a reference genome, making comparisons between studies’ findings difficult. The recent publication of a full chromosome-anchored wheat genome assembly should allow for better curation of MTAs uncovered by GWAS and linkage mapping studies.

The present study sought to perform GWAS in a panel of elite soft winter wheat lines sourced from breeding programs in the Eastern and Midwestern United States. Genotyping was performed via genotyping-by-sequencing (GBS), using genetic maps and anchoring SNPs to the Chinese Spring reference sequence International Wheat Genome Sequencing Consortium (IWGSC) RefSeq v1.0 [34], allowing for determination of both physical and genetic positions for identified MTAs. In addition, targeted genotypic assays were used to interrogate loci which are known to exert major effects on many traits of agronomic importance.

## Materials and methods

### Germplasm selection

Germplasm selection was performed as detailed in Ward et al., 2019 [35]. The study was conducted over two years, and included a total of 329 genotypes (S1 Table). Of these, 41 genotypes were tested across both years. Of the remaining genotypes, half (144) were tested only in the first year, and the other half (144) were tested only in the second year. Within each year, genotypes were sourced from breeding programs in Illinois (31), Kentucky (30), Missouri (2), and Virginia (122). Five checks were included in the study: ‘Bess’, ‘Branson’, IL00-8530, ‘Roane’, and ‘Shirley’. With the exception of checks and several older cultivars, the majority of genotypes were either F_4_ or F_5_ filial generation.

### Phenotyping

#### Experimental design and data collection

Experimental design, field management practices, and phenotypic data collection were performed as detailed in Ward et al., 2019 [35]. Briefly, the experiment was planted in a total of four environments (two locations in two years) in the 2013–14 and 2014–15 winter wheat growing seasons. Within each year, trials were planted at Kentland Farm near Blacksburg, Virginia (Guernsey/Hayter silt loams, 37.1965° N, 80.5718° W, 531 m elevation) and the Eastern Virginia Agricultural Research and Extension Center in Warsaw, Virginia (Kempsville sandy loam, 37.9879° N, 76.7770° W, 40 m elevation). A generalized randomized complete block design (GRCBD) with two replications was used in each environment. Data was collected on 14 traits, including flag leaf stay green duration (FLSG), grains per square meter (GSQM), grain weight (GW), heading date (HD), plant height (HT), physiological maturity date (MAT), normalized-difference vegetation index (NDVI) at Zadok’s growth stage 25 [36], whole-grain protein content (PROT), seeds per head (SPH); spikes per square meter (SSQM), whole-grain starch content (STARCH), thousand kernel weight (TKW), test weight (TWT), and grain yield (YLD). S2 Table summarizes all phenotypic traits, and lists their abbreviations, units of measure, and trait ontologies.

#### Spatial corrections

For each individual trait/environment combination, an *ad-hoc* correction for field heterogeneity was performed using two-dimensional tensor product penalized B-splines [37], implemented in a mixed model framework in the R [38] package ‘SpATS’ [39]. The package default parameters were used to fit cubic splines with quadratic penalization functions in both row and column dimensions. For a particular environment with plots arranged in *m* rows and *n* columns, the number of knots used to fit splines was set to (⌊*m*⌋/2) − 1 and (⌊*n*⌋/2) − 1, respectively. Fitted values generated by the SpATS model were used for subsequent modeling of phenotypes across environments, as described in the section below.

Heritability for each trait/environment combination was estimated using the method of Cullis, et al. [40]:

hg2=1-Att2σG2# (1)

Where the generalized heritability ($h_{g}^{2}$) is a function of the average pairwise prediction error between pairs of genotypes within an environment (*A*_*tt*_), and the genotypic variance ($\sigma_{G}^{2}$). Heritability estimates were calculated from the models fit by the SpATS package, and were compared against the heritability estimates generated by a baseline model:

Yi=μ+Gi+εi# (2)

Where phenotypic response (*Y*_*i*_) is a function of the within-environment mean (μ), the fixed effect of the *i*th genotype (*G*_*i*_), and residual error (*ε*_*i*_).

#### Modelling of phenotypes across environments

Each location/year combination was considered as a unique environment in order to model phenotypic response across environments. For each trait, the following random effects ANOVA model was fit in R using the ‘lme4’ package [41]:

Yijk=μ+Gi+Ej+Rk(Ej)+GEij+εijk# (3)

Where the phenotypic response (*Y*_*ijk*_) is a function of the overall mean (*μ*) and the random effects of the ith genotype (*G*_*i*_), the kth replication (*R*_*k*_) nested within the jth environment (*E*_*j*_), the genotype-environment interaction (*GE*_*ij*_) and the residual error (*ε*_*ijk*_). Genotypic best-linear unbiased predictors (BLUPs) were calculated for use as the phenotypic input for the subsequent GWAS analyses.

### Genotyping

#### Genotyping-by-sequencing

Genomic DNA was isolated from fresh green seedling leaf tissue using an LGC Genomics Oktopure robotic extraction platform with sbeadex magnetic microparticle reagent kits. Genotyping-by-sequencing was performed using an Illumina HiSeq 2500 following a double digest of genomic DNA using the restriction enzymes *PstI* and *MseI*, using the protocol of Poland et al., 2012 [42]. SNP calling was performed using TASSEL-GBS v5.2.43 [43,44]. The Burrows-Wheeler Aligner [45] v0.7.17-r1188 was used to align Illumina-generated short reads to the Chinese Spring IWGSC RefSeq v1.0 wheat reference sequence. The raw genotypic data was filtered to retain only biallelic SNPs, and to remove SNPs with missing data frequencies > 50%, mean sequencing depth < 2, heterozygous call frequencies > 15%, or minor allele frequency < 5%. SNPs that aligned to unmapped contigs were removed. Genotypes containing > 85% missing data were removed. Missing data was then imputed using Beagle v4.1 with default settings [46]. After imputation, the genotypic data was filtered a second time to remove SNPs with minor allele frequency < 5% or heterozygous call frequency < 15%. PLINK 1.9 [47] was used to remove all but one SNP in groups of SNPs in perfect linkage disequilibrium (LD; r^2^ = 1). After filtering, 29,949 SNPs and 322 genotypes remained for further analysis.

#### Assays for polymorphisms of major effect

Several genes and polymorphisms of major effect were assayed using LGC Genomics KASPar SNP assays. All included assays are listed with primer sequences in S3A Table. Briefly, they included assays for the 1RS:1AL and 1RS:1BL translocations from rye (*Secale cereal* L.), polymorphisms within the *Ppd-A1*, *Ppd-B1*, and *Ppd-D1* photoperiod sensitivity genes located on chromosomes 2A, 2B, and 2B respectively, the *Rht-B1* and *Rht-D1* dwarfing genes located on chromosomes 4B and 4D respectively, and polymorphisms of the *Vrn-A1* and *Vrn-B1* genes located on chromosomes 5A and 5B.

KASP assays for polymorphisms within exon 4 and exon 7 of *Vrn-A1* were included in this study. The molecular mechanism behind *Vrn-A1*’s effects on vernalization requirement remains contested. Chen et al. [48] proposed that a SNP occurring in exon 4 of *Vrn-A1* was the causal locus differentiating between the *Vrn-A1a* short vernalization requirement allele present in the cultivar Jagger and the *Vrn-A1b* long vernalization requirement allele present in the cultivar ‘2174’. Díaz et al. [49] later proposed that the differences between *Vrn-A1a* and *Vrn-A1b* vernalization requirements are due to *Vrn-A1* copy number variations. Li et al. [50] subsequently reported that although Jagger contains two copies of *Vrn-A1*, whereas ‘2174’ contains three copies of the gene, differences in vernalization requirements between the two cultivars are in fact due to structural differences, particularly due to a SNP located in exon 7, converting the 180^th^ amino acid residue from alanine in *Vrn-A1a* to valine in *Vrn-A1b*. Finally, Kippes et al. [51] proposed that the effects of *Vrn-A1* arise from the product of gene *VRN-D4* disrupting the binding of the RNA-binding repressor *TaGRP2* to *Vrn-A1*. The KASP assays for *Vrn-A1* used in this study have been shown to be suggestive, but not perfect predictors of vernalization requirement due to *Vrn-A1* alleles.

In addition, assays for the *Sr36* stem rust (*Puccinia graminis* Pers.) resistance gene, and the sucrose-synthase gene *TaSus2-2B*, which affects kernel weight, were included. Gene *Sr36* is located on a 2G:2B alien translocation originating from *Triticum timopheevi* [Zhuk.] Zhuk. (A^m^A^m^GG) [52,53]. Gene *TaSus2-2B* is located on the short arm of chromosome 2B, and is one of the three sucrose synthase *Sus2* orthologs located on chromosomes 2A, 2B, and 2D. Two common haplotypes for *TaSus2-2B* include *Hap-H* (high seed weight) residing on the 2G:2B translocation, and *Hap-L* (low seed weight) [13]. While there is no evidence to suggest that *TaSus2-2B* was inherited from *T*. *timopheevi*, Cabrera et al. found that *TaSus2-2B* alleles were in perfect LD with alleles of the microsatellite marker *Xwmc477* [54], which itself was previously found to be in perfect LD with *Sr36* [55].

### Population structure and linkage disequilibrium

Prior to performing GWAS, population structure was examined via principle component analysis (PCA) of the filtered and imputed genotypic data using the SNPRelate package [56] in R. Linkage disequilibrium (r^2^) was estimated between all intrachromosomal pairs of SNPs up to a physical distance of 10Mb using PLINK 1.9. The pairwise r^2^ estimates were then sorted into bins from 10^2^ to 10^7^ base pairs, with an exponent interval size of 0.1. The mean r^2^ value was calculated for each bin, and then plotted against physical distance, with a second-degree locally-weighted scatterplot smoothing (LOESS) curve fit to the data [57]. Separate LOESS curves were fit to LD data from each genome, and from each chromosome. Mean r^2^ values equal to or greater than 0.2 were considered significant LD. In addition, LD was calculated between the KASP *Sr36* assay and all other SNPs present on chromosome 2B. Finally, the fixation index (*F*_*ST*_) was calculated for each SNP using Weir and Cockerham’s estimator [58], with two subpopulations defined by the presence or absence of *Sr36* as defined by the KASP assay results. All *F*_*ST*_-related calculations were performed using VCFtools v0.1.17 [59].

### Genome-wide association analysis

For each trait, single-locus mixed linear model genome-wide association analyses were performed with the Genome-Wide Complex Trait Analysis (GCTA) software [60], using a leave-one-chromosome-out (LOCO) method in which a separate genetic relationship matrix (GRM) is estimated from SNP data for each chromosome. Specifically, the LOCO approach entails excluding all SNPs located on the chromosome of the SNP undergoing testing when estimating the GRM. Adjusted p-values for each SNP were calculated using the method of Benjamini and Hochberg [61], and SNPs with adjusted p-values below 0.05 were considered significant.

In addition, GWAS was performed for each trait using the Fixed and Random Model Circulating Probability Unification (FarmCPU) model [21], using the R package ‘FarmCPUpp’ [62]. The same significance threshold applied in the single-locus tests was used for the FarmCPU results. To enhance our confidence in significant MTAs identified by FarmCPU, we implemented a bootstrapping method utilized by Wallace, et al. [63], in which 10% of the phenotypic observations were randomly replaced with missing data for a total of 100 runs of the model. Subsequently, for each trait the resample model inclusion probability (RMIP) [64] was calculated for each SNP by determining the fraction of bootstraps in which its adjusted p-value exceeded the significance threshold. The value 0.1 was chosen as a lower threshold for the RMIP as it coincided with the point of inflection in the RMIP density curves (data not shown). For each model, the first four principal components were included to model population structure, based upon visual examination of the scree plot and cumulative sum plots for variance explained by each PC (S1 Fig). Approximate genetic positions were calculated for each MTA using a set of *PstI-MspI* GBS markers generated from 88 doubled haploid lines derived from the synthetic W7894 × Opata M85 (SynOp) cross [65], aligned to the Chinese Spring IWGSC RefSeq v1.0 wheat reference sequence [34]. The R package ‘MonoPoly’ [66] was used to fit a monotonically increasing spline to each chromosome of the SynOp genetic map, allowing for the estimation of genetic positions corresponding to MTA physical positions.

### Candidate genes and translation effects

Haplotype blocks surrounding each significant MTA were identified using the method of Gabriel et al. [67] by running the—blocks command in PLINK 1.9. Some significant MTAs did not reside within any larger haplotype block, while some haplotype blocks contained multiple significant MTAs. Subsequently, all genes overlapping significant MTAs and associated haplotype blocks were identified using the IWGSC v1.1 RefSeq annotation [34] with functional annotations from the IWGSC v1.0 annotation. In addition, Ensembl identifiers were retrieved for all genes overlapping with significant SNPs or haplotype blocks. A table of wheat genes with trEMBL or Swissprot-generated protein annotations in UniProt was downloaded and used to identify all wheat genes with predicted functions located within 1Mb of each significant MTA. The Ensembl Variant Effect Predictor [68] was then used to classify significant SNPs as being either intergenic, intronic, exonic, or upstream/downstream proximal variants. The predicted allele substitution effects of exonic SNPs on protein translation were classified as synonymous, missense, or nonsense. For gene-proximal SNPs, the distance to the closest gene was recorded.

## Results

### Trait heritability and correlations

The use of spatially-corrected data tended to drastically increase generalized heritability within each environment. The trait GW had the lowest mean generalized heritability, averaging 0.2 across environments for raw, uncorrected data, and 0.33 after performing spatial corrections (Table 1). The trait TKW had the highest mean generalized heritability when using uncorrected data $\left(h_{G}^{2}=0.95\right)$, though heritability was slightly decreased when using spatially-corrected data $\left(h_{G}^{2}=0.9\right)$. When spatially-corrected data was used, TWT had the highest average heritability $\left(h_{G}^{2}=0.95\right)$. YLD had a moderate mean, across-environment heritability of 0.57 when calculated using uncorrected data, though this increased to 0.81 when using spatially-corrected data. Pearson correlation coefficients were calculated for each pair of traits using the phenotypic BLUPs (Table 2). Phenological traits (HD, FLSG, and MAT) all displayed a high degree of intercorrelation. This was also generally the case for traits relating to grain size and density per unit area (TKW, GSQM, SSQM, and SPH). For instance, the traits GSQM and TKW demonstrated a strong negative correlation (-0.66), as predicted due to trait compensation effects. Of these four traits, the weakest correlation was between TKW and SPH (-0.24). Critically, only weak correlation was observed between traits relating to grain size/density and phenological traits, suggesting that these two classes of traits could be improved independently. YLD was most highly correlated with the traits GW and MAT (positive), and grain protein (negative). The negative correlation between yield and grain protein content has been well documented in the past (e.g. [69–71]).

**Table 1:** Trait descriptive statistics and generalized heritability estimates calculated using raw plot values and spatially-adjusted values.

		Descriptive Statistics				Generalized Heritability									
						Raw Plot Values					Spatially-Adjusted Values				
Trait ^a^	Units	min	mean	max	SD	14Bb	14War	15Bb	15War	mean	14Bb	14War	15Bb	15War	mean
FLSG	days	21.0	28.7	38.0	2.76	0.26	0.80	0.75	0.55	0.59	0.55	0.83	0.76	0.75	0.72
GSQM	Grains m-2	8460	1.85E+04	3.13E+04	3277	0.62	0.50	0.51	0.58	0.55	0.71	0.51	0.54	0.67	0.61
GW	g dwt m-1 row	47.68	96.64	157.9	16.53	0.19	0.14	0.44	0.05	0.20	0.43	0.16	0.47	0.25	0.33
HD	Julian days (Jan1)	121	128	136	3.21	0.75	0.94	0.95	0.96	0.90	0.82	0.95	0.96	0.97	0.92
HT	cm	59	85	119	9.3	0.85	0.85	0.84	0.86	0.85	0.92	0.88	0.88	0.87	0.89
MAT	Julian days (Jan1)	151	159	171	4.72	0.83	0.87	0.86	0.76	0.83	0.89	0.91	0.89	0.9	0.90
NDVI	-	0.26	0.54	0.75	0.08	0.06	0.39	0.41	0.41	0.32	0.70	0.75	0.68	0.64	0.69
PROT	%	9.67	12.3	16.0	1.01	0.66	0.38	0.63	0.74	0.60	0.67	0.40	0.71	0.78	0.64
SPH	count	8.54	21.9	33.3	3.04	0.86	0.82	0.77	0.81	0.82	0.85	0.82	0.75	0.84	0.82
SSQM	Spikes m-2	459.3	853.0	1485	161.2	0.60	0.62	0.45	0.52	0.55	0.65	0.63	0.50	0.63	0.60
STARCH	%	46.88	52.51	56.49	1.410	0.46	0.71	0.19	0.69	0.51	0.80	0.72	0.73	0.83	0.77
TKW	grams	24.1	34.6	91.6	3.98	0.96	0.97	0.94	0.93	0.95	0.97	0.98	0.69	0.97	0.90
TWT	g L-1	652.6	759.0	810.9	19.70	0.96	0.92	0.91	0.93	0.93	0.98	0.96	0.90	0.95	0.95
YLD	kg ha-1	3579	6627	9053	1027	0.63	0.45	0.73	0.46	0.57	0.84	0.81	0.78	0.81	0.81

14Bb Blacksburg, VA 2014; 14War Warsaw, VA 2014; 15Bb Blacksburg, VA 2015; 15War Warsaw, VA 2015

^**a**^ FLSG flag leaf stay green; GSQM grains per square meter; GW grain weight; HD heading date; HT plant height; MAT physiological maturity date; NDVI normalized-difference vegetation index at Zadok’s GS25; PROT wet chemistry-validated whole-grain protein content; SPH seeds per head; SSQM spikes per square meter; STARCH whole-grain starch content; TKW thousand kernel weight; TWT test weight; YLD grain yield

**Table 2:** Phenotypic correlations among traits calculated using the across-environment genotype BLUPs.

	**FLSG**	**GSQM**	**GW**	**HD**	**HT**	**MAT**	**NDVI**	**PROT**	**SPH**	**SSQM**	**STARCH**	**TKW**	**TWT**	**YLD**
**FLSG**	1													
**GSQM**	0.01	1												
**GW**	0.27*	0.64*	1											
**HD**	-0.43*	0.14*	-0.06	1										
**HT**	-0.18*	-0.16*	-0.21*	0.15*	1									
**MAT**	0.18*	0.26*	0.19*	0.71*	-0.07	1								
**NDVI**	0.05	0.05	0.08	0.12	0.13	0.10	1							
**PROT**	0.07	-0.41*	-0.39*	-0.15*	0.11	-0.21*	0.15*	1						
**SPH**	0	0.48*	0.40*	0.12	0.02	0.21*	-0.20*	-0.39*	1					
**SSQM**	0.02	0.53*	0.24*	0.02	-0.18*	0.05	0.25*	-0.01	-0.48*	1				
**STARCH**	0.20*	0.30*	0.32*	-0.04	-0.23*	0.19*	-0.12	-0.62*	0.29*	0.01	1			
**TKW**	0.25*	-0.66*	0.14	-0.24*	-0.01	-0.13	0.01	0.15*	-0.24*	-0.43*	-0.07	1		
**TWT**	0.04	-0.22*	-0.19*	-0.28*	0.18*	-0.31*	-0.08	0.25*	-0.20*	-0.02	-0.25*	0.10	1	
**YLD**	0.32*	0.36*	0.58*	0.18*	-0.08	0.48*	0.13	-0.45*	0.33*	0.03	0.41*	0.11	-0.20*	1

FLSG flag leaf stay green; GSQM grains per square meter; GW grain weight; HD heading date; HT plant height; MAT physiological maturity date; NDVI normalized-difference vegetation index at Zadok’s GS25; PROT wet chemistry-validated whole-grain protein content; SPH seeds per head; SSQM spikes per square meter; STARCH whole-grain starch content; TKW thousand kernel weight; TWT test weight; YLD grain yield

* Correlation significant at the 0.01 level

### Polymorphisms of major effect

An estimation of allele effects for the KASP markers assaying genes of known function revealed that the stem rust resistance gene *Sr36* and the sucrose-synthase gene *TaSus2-2B* produced many significant differences among genotypes for multiple traits (S3B Table). These two genes also consistently produced significant differences of similar magnitudes for the same traits. The presence of the *Rht-B1b* and *Rht-D1b* dwarfing alleles produced significant effects of opposite signs for many traits, including SSQM, TKW, and TWT. Effects were also of opposite signs for HT and YLD, though in both of these cases the effects of *Rht-B1b* were not significant. The *Vrn-A1* exon 4 polymorphism and *Vrn-B1* polymorphism each exerted significant effects on six traits, while the *Vrn-A1* exon 7 polymorphism produced significant effects on three traits. However, these polymorphisms occurred at low frequencies (0.14, 0.03, and 0.07 for *Vrn-A1* exon 4, *Vrn-A1* exon7, and *Vrn-B1* respectively). Of the three *Ppd* photoperiod response genes assayed, *Ppd-D1* produced significant effects on five traits, while also occurring at a high frequency (0.69), while *Ppd-B1* produced significant effects on four traits, occurring at a lower frequency (0.26). *Ppd-A1* occurred at a high frequency, but only produced a significant effect on the trait GW. Finally, the 1RS:1AL and 1RS:1BL translocations produced significant effects on five and four traits, respectively, but occurred at relatively low frequencies of 0.07 and 0.19. Despite these significant effects on phenotype, none of the loci of major effect assayed with KASP markers were classified as significant in the GWAS. Some potential reasons for these findings will be discussed below.

### Population structure and linkage disequilibrium

**Fig 1:**
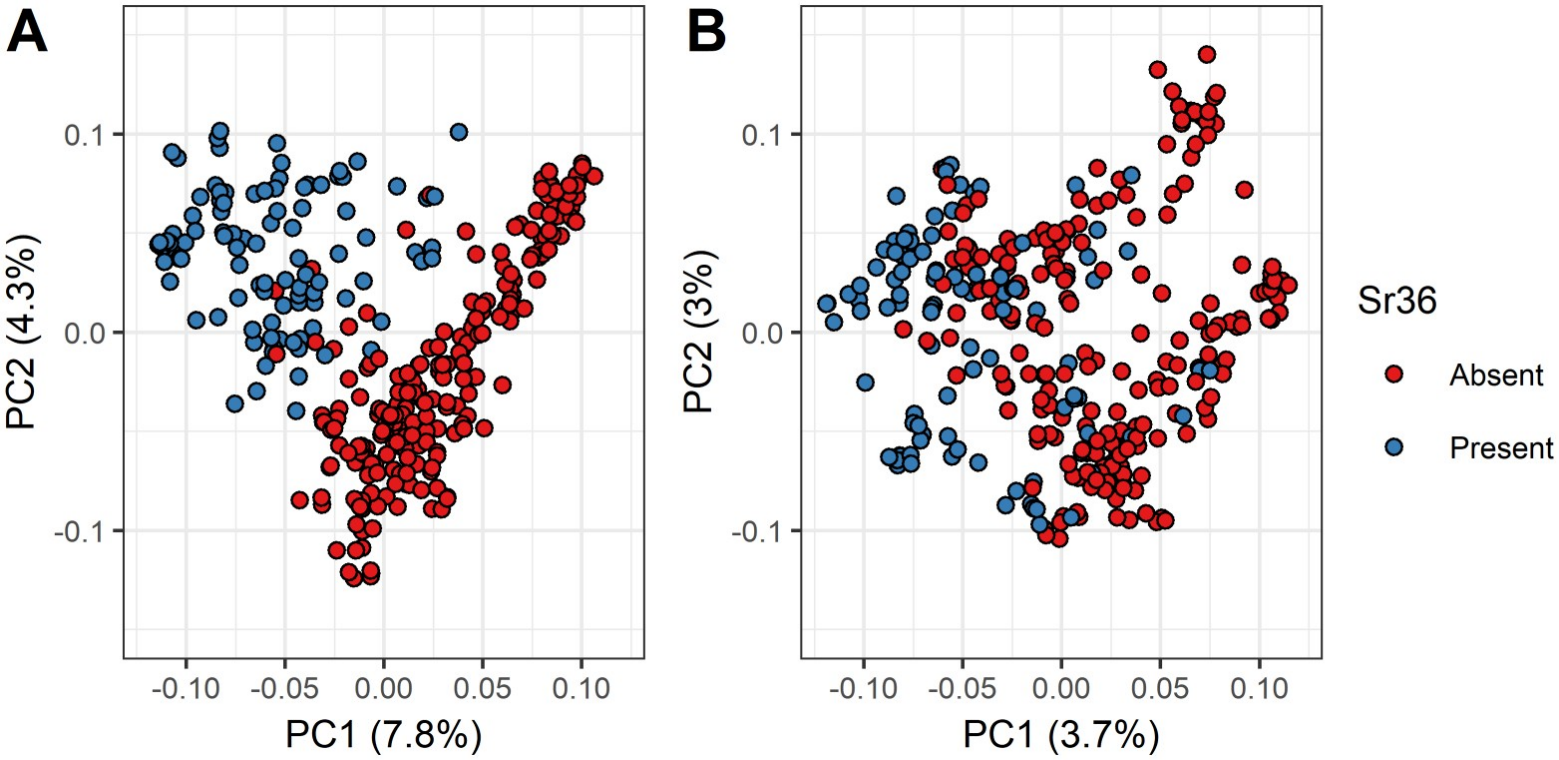
Biplots of genotypic principal components. The first and second principal components of the SNP matrix were plotted against each other, using (A) all SNPs that passed initial filtering parameters, and (B) a thinned subset of SNPs selected to be in approximate linkage equilibrium, so that no pair of SNPs displayed significant LD (r^2^ > 0.2). The percent of the total genotypic variance explained is listed on each axis. Genotypes are divided into two groups based on the presence of absence of the *Sr36* stem rust resistance gene and underlying 2G:2B translocation as determined by KASP assay.

Principle component analysis of the processed GBS SNP data revealed substantial admixture among genotypes, with the first principle component only explaining 7.84% of the total genotypic variance (S1 Fig). Lines included in the panel formed two distinct clusters in the biplot of the first two principle components. These two clusters were largely delineated by the presence or absence of the 2G:2B translocation as determined by the *Sr36* KASP assay (Fig 1A; similar results for *TaSus2-2B* not shown). In contrast, neither the 1BL:1RS nor the 1AL:1RS alien translocations produced any discernable clustering of genotypes (data not shown). When the SNP data was thinned to remove SNPs in high LD with each other (i.e. limiting the maximum pairwise LD between SNPs to r^2^ = 0.2), the population stratifying effects of *Sr36* and the underlying 2G:2B translocation were removed, and the first principle component explained 3.68% of variation (Fig 1B).

**Fig 2:**
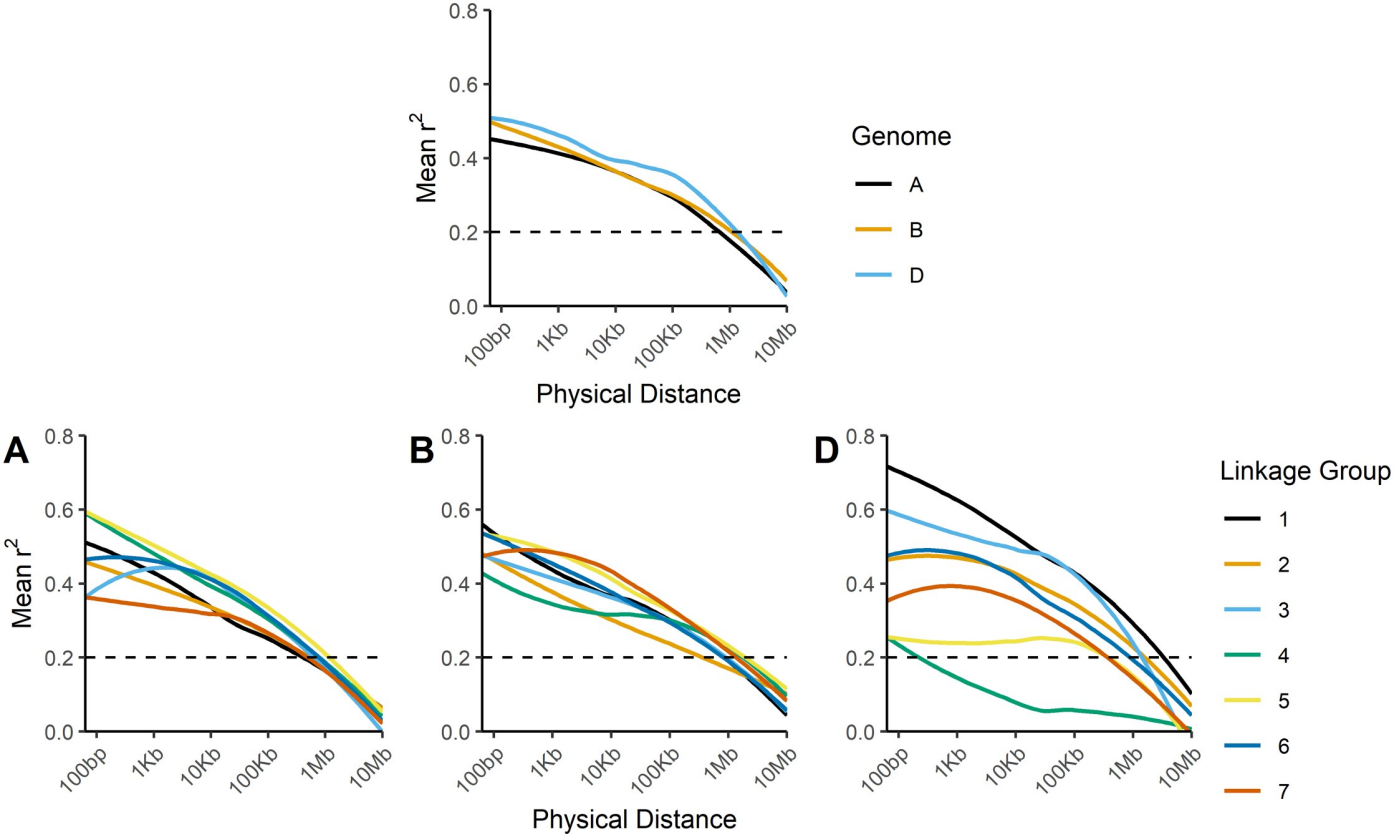
Linkage disequilibrium by genome and chromosome. LOESS regressions of mean r^2^ between pairs of SNPs vs. physical distance, pooled for each genome (top row), and for each of the seven chromosomes present in the A, B, and D genomes (bottom row).

**Fig 3:**
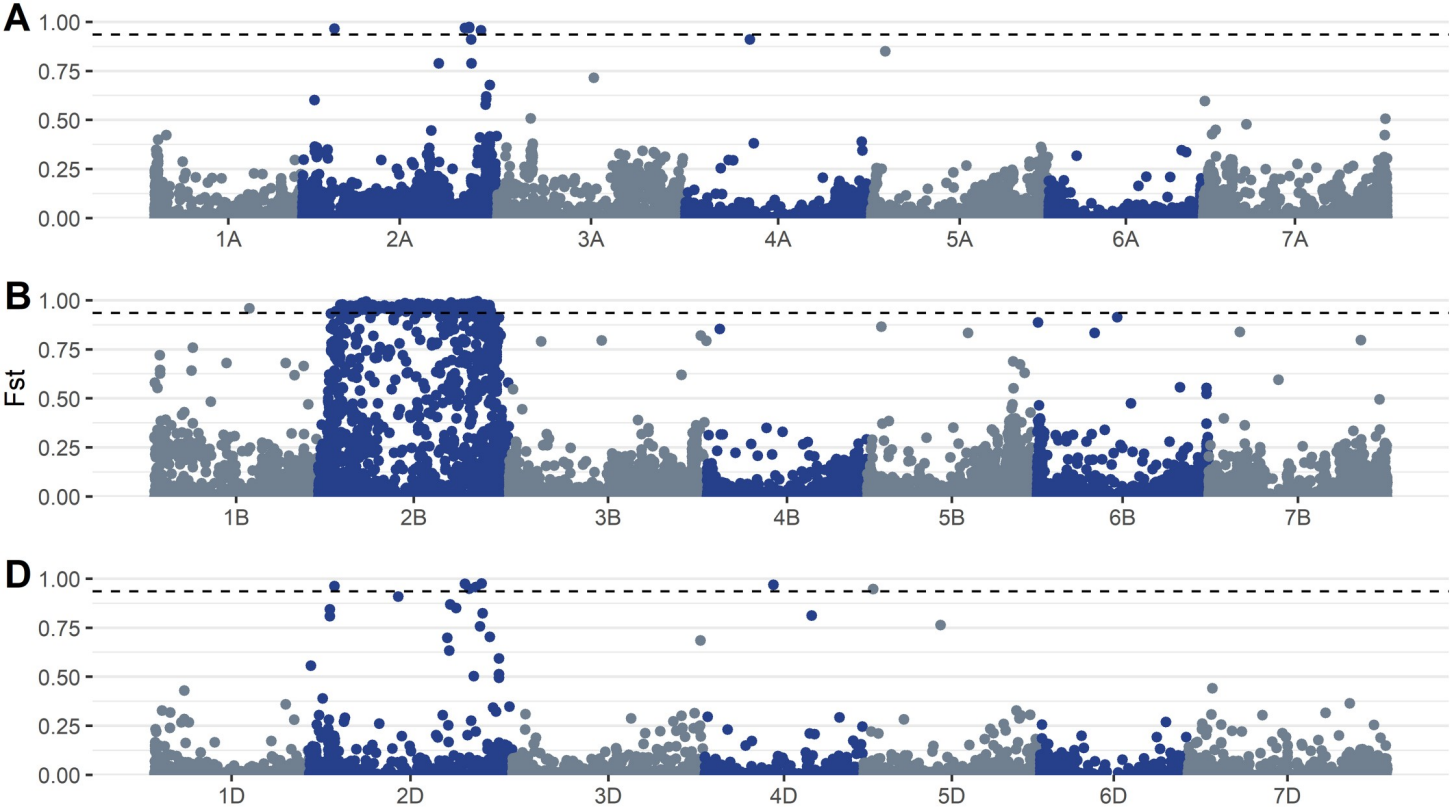
Genome-wide *F*_*ST*_ scan. *F*_*ST*_ values were estimated for each SNP in the A genome (top row), B genome (middle row), and D genome (bottom row). The dashed line signifies the 99^th^ percentile of *F*_*ST*_ values for all SNPs.

**Fig 4:**
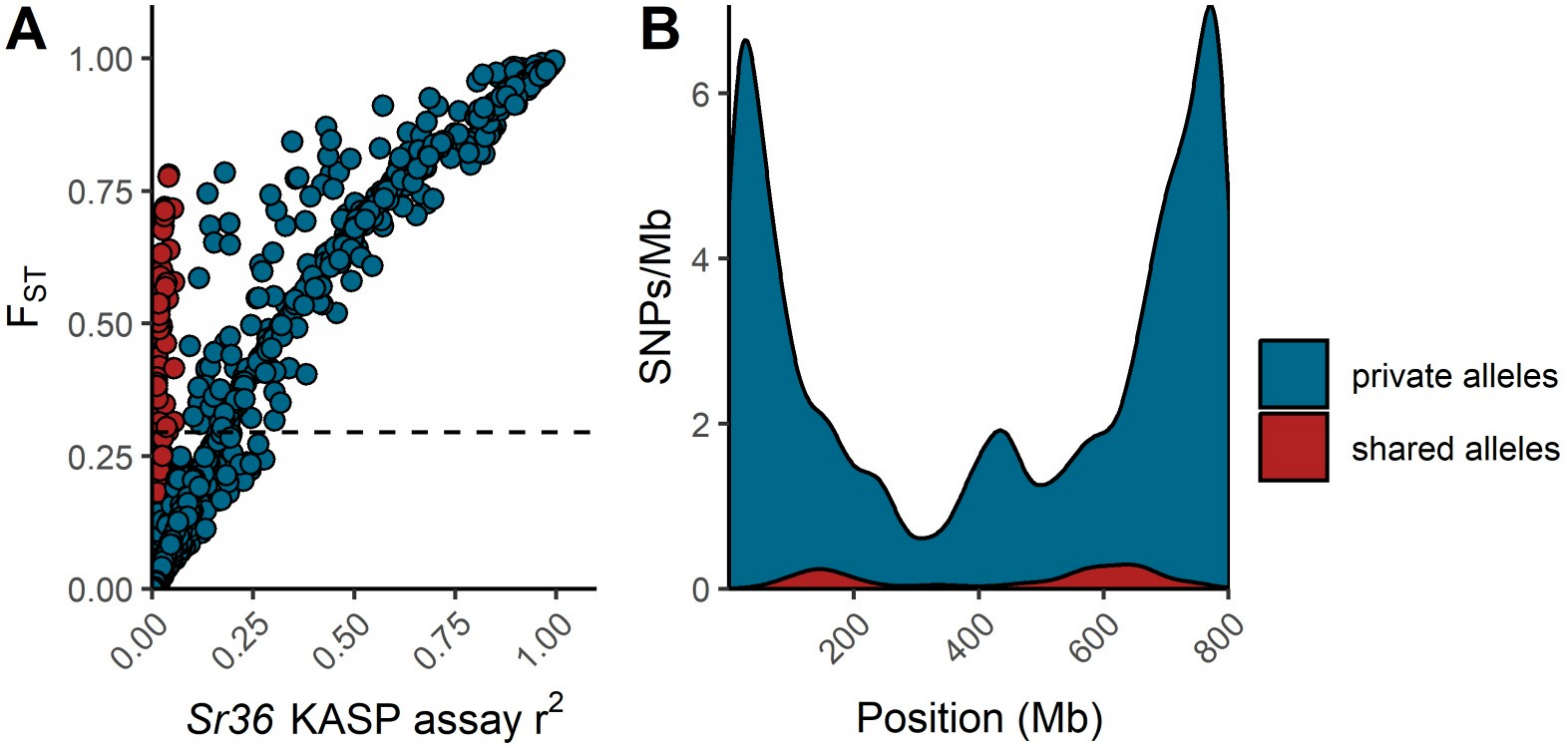
*Triticum timopheevi* private and shared alleles on chromosome 2B. (A) *F*_*ST*_ plotted against r^2^ with the KASP *Sr36* assay for all SNPs on chromosome 2B. Two clusters of SNPs were manually differentiated—those with putative *Triticum timopheevi* private alleles for which *F*_*ST*_ and r^2^ values show a general linear relationship (blue), and those with putative *Triticum timopheevi*/*Triticum aestivum* shared alleles for which *F*_*ST*_ shows no relationship with r^2^ (red). Horizontal dashed line represents the 99^th^ percentile of genome-wide *F*_*ST*_ values, excluding SNPs on chromosomes 2A, 2B, and 2D. (B) Density plots of both classes of SNPs across chromosome 2B.

Linkage disequilibrium decay plots demonstrated that LD decayed below significant levels (r^2^ < 0.2) at distances of approximately 1Mb (Fig 2). LD decay was highly similar between chromosomes, with the exception of chromosomes located on the D genome, which exhibited greater variation in LD decay patterns. This may simply be due to differences in SNP density among the genomes. As SNP density was much lower in the D genome (S2 Fig), many D genome chromosomes had far fewer pairs of SNPs in close proximity to each other. This likely inflated the variance of LD estimates at short distances. The genome-wide *F*_*ST*_ scan following the splitting of the overall panel based on the result of the *Sr36* KASP assay demonstrated that the 2G:2B translocation formed a large block of high-*F*_*ST*_ SNPs spanning almost all of chromosome 2B (Fig 3). In addition, an enrichment of high-*F*_*ST*_ SNPs on chromosomes 2A and 2D suggested misalignment of SNPs among the group 2 homeologous chromosomes. Further examination of *F*_*ST*_ values on chromosome 2B indicated a general linear relationship between *F*_*ST*_ and the value of r^2^ measured against the *Sr36* KASP marker for most SNPs, with a minority of SNPs exhibiting high *F*_*ST*_ with little or no corresponding LD with the *Sr36* KASP marker (Fig 4A). These SNPs were distributed throughout the chromosome, though SNP density tended to be higher in telomeric regions. In addition, SNPs with *T*. *timopheevi* private alleles were far more abundant than SNPs with shared alleles. (Fig 4B).

### Genome-wide association studies

In general, the single-locus model implemented in GCTA identified far fewer significant MTAs than FarmCPU. In total, GCTA identified 44 significant MTAs at the 0.05 FDR significance threshold. However, many of these were located within high-LD blocks and, therefore, clustered at the same putative underlying QTLs. Discounting those SNPs believed to co-localize to identical QTLs yielded nine unique MTAs for HD, MAT, SSQM, TKW, and TWT (Table 3). One SNP, located at 58,633,321bp on chromosome 7D, was pleiotropic for the traits HD and MAT, and hence the nine significant MTAs represented eight putative QTLs. In contrast, FarmCPU identified 108 MTAs at the same significance threshold. Removing those SNPs with RMIPs below 0.1 yielded a total of 74 significant MTAs for the traits FLSG, GSQM, GW, HD, HT, MAT, NDVI, PROT, SPH, SSQM, STARCH, TKW, TWT, and YLD (Table 4). After filtering by the RMIP threshold, the remaining significant FarmCPU MTAs exhibited an approximate uniform distribution for RMIP values. A significant MTA affecting the trait TWT located on the long arm of 6A had the highest RMIP value of 0.99. Since FarmCPU fits significant MTAs as covariates based partially upon LD estimates, the MTAs identified by this model for a given trait all reside within different putative QTLs. However, several MTAs did occur for different traits associated with the same SNP, or else clustered in high-LD regions with pleiotropic effects, and hence the 74 total MTAs are represented by 67 putative QTLs, with five QTLs affecting multiple traits. Of the eight putative QTLs identified by GCTA, four were also identified by FarmCPU. Examination of the Manhattan plots and uniform quantile-quantile plots of the p-values produced by the single-locus model (S1 File) and FarmCPU (S2 File) demonstrated adequate control of p-value inflation for most traits, with FarmCPU properly detecting and correcting for LD between SNPs co-located on QTLs.

**Table 3:** Significant marker-trait associations identified by the GCTA leave-one-chromosome-out method.

Trait^a^	Chr	Pos^b^	cM^c^	Alleles	MAF	P-value	Effect	Units	References
HD	1B	50989662	52.8	G\A	0.29	1.00E-06	0.533	Julian days	[72,73]
HD	6A	150099119	109.5	A\T	0.17	1.52E-05	-0.507	Julian days	[72]
HD	6A	542256574	128.5	T\C	0.34	3.39E-05	-0.417	Julian days	[74,75]
HD	7D	58633321^A^	84.6	A\G	0.33	3.70E-10	-0.634	Julian days	
MAT	2B	3266917	0	C\A	0.15	2.35E-05	-0.527	Julian days	
MAT	7D	58633321^A^	84.6	A\G	0.33	5.71E-07	-0.495	Julian days	
SSQM	3A	6788503	12.5	C\T	0.46	1.56E-06	16.0	Spikes m^-2^	
TKW	1A	40498783	68.0	G\T	0.09	1.62E-06	1.25	grams	[75,76]
TWT	1A	583992305	218.8	T\C	0.23	9.33E-07	-3.39	g L^-1^	

Chr chromosome; Pos physical position; cM genetic position in centiMorgans; Alleles major allele listed first, minor allele second, favorable allele (if any) underlined; MAF minor allele frequency; Effect mean difference in trait value caused by substituting minor allele for major allele

^a^ HD heading date; MAT physiological maturity date; SSQM spikes per square meter; TKW thousand-kernel weight; TWT test weight

^b^ Superscript letters indicate QTLs with pleiotropic effects

^**c**^ Genetic position estimated from synthetic W7894 × Opata M85 GBS genetic map aligned to Chinese Spring IWGSC RefSeq v1.0 wheat reference sequence

**Table 4:** Significant marker-trait associations identified by the FarmCPU algorithm.

Trait^a^	Chr	Pos^b^	cM^c^	Alleles	MAF	P-value	Effect	Units	RMIP	References
FLSG	2A	664114222	101.7	G\C	0.26	4.30E-06	0.198	days	0.12	
FLSG	3A	660434027	145.9	T\C	0.07	6.09E-09	0.553	days	0.61	
FLSG	4D	389572522	82.9	G\A	0.12	3.37E-07	-0.278	days	0.19	
FLSG	6A	13888173	26.4	A\C	0.06	3.16E-07	0.487	days	0.33	
FLSG	7A	727690323	222.7	G\A	0.16	3.65E-06	-0.217	days	0.14	
FLSG	7B	59821499	61.8	C\T	0.37	3.84E-07	0.229	days	0.12	
FLSG	7B	64138826	62.4	A\G	0.17	7.28E-06	0.235	days	0.37	
GSQM	1A	103223278	85.0	A\T	0.43	1.25E-08	-309	Grains m^-2^	0.37	
GSQM	5B	396479359	104.4	C\T	0.46	5.71E-06	-246	Grains m^-2^	0.14	
GSQM	7A	673460466^A^	151.6	C\T	0.46	1.89E-07	256	Grains m^-2^	0.72	
GSQM	7B	444463299^B^	82.4	A\G	0.06	2.03E-06	-650	Grains m^-2^	0.25	
GSQM	7D	117245582	131.0	T\G	0.08	3.38E-06	450	Grains m^-2^	0.17	
GW	2A	768420387	162.2	G\C	0.38	9.84E-06	0.467	g dwt m^-1^ row	0.22	
GW	3A	704973519	192.7	A\G	0.12	4.29E-06	0.441	g dwt m^-1^ row	0.16	
GW	5B	34551165	61.2	C\T	0.38	7.62E-08	-0.494	g dwt m^-1^ row	0.31	
GW	7A	55399749	61.5	A\G	0.17	8.56E-08	0.651	g dwt m^-1^ row	0.23	
HD	1B	50989662	52.8	A\G	0.29	1.04E-09	0.438	Julian days	0.31	[72,73]
HD	2D	35084672	46.8	G\A	0.12	1.30E-07	-0.415	Julian days	0.66	
HD	5B	710175294	312.9	A\C	0.05	9.92E-07	0.797	Julian days	0.16	
HD	6A	449693253^C^	112.4	C\T	0.34	2.01E-06	0.317	Julian days	0.58	[72]
HD	7B	97678492	65.4	G\A	0.05	1.43E-06	-0.862	Julian days	0.38	[30]
HD	7D	58633321^D^	84.6	A\G	0.32	3.00E-13	-0.517	Julian days	0.78	[72]
HD	7D	553154931	173.3	C\T	0.4	1.57E-06	-0.314	Julian days	0.22	[73]
HT	1A	13666754	29.4	T\G	0.06	4.91E-08	-1.83	cm	0.38	[77]
HT	2A	764324892	158.4	T\A	0.22	5.36E-06	-1.05	cm	0.40	[78]
HT	2D	181663205	100.4	C\A	0.49	1.13E-07	2.21	cm	0.58	
HT	6B	706327428	160.6	A\G	0.07	5.73E-07	1.53	cm	0.31	[78]
MAT	2A	7231537	3.0	G\C	0.4	2.92E-10	0.460	Julian days	0.36	
MAT	2B	4475209	1.8	T\A	0.48	3.55E-08	-0.315	Julian days	0.25	
MAT	3B	737436815	178.2	G\A	0.38	2.77E-09	-0.380	Julian days	0.73	
MAT	7B	232267370	75.5	G\A	0.11	3.52E-07	-0.486	Julian days	0.17	
MAT	7D	58633321^D^	84.6	A\G	0.32	1.27E-07	-0.364	Julian days	0.47	[72]
NDVI	1A	251018060	88.4	C\T	0.07	8.85E-07	-0.00799	-	0.79	
NDVI	7A	127596189	104.1	T\C	0.11	7.94E-08	-0.00481	-	0.11	
PROT	1A	465502281	103.5	A\G	0.36	1.13E-06	0.0641	%	0.31	
PROT	5A	594957276	119.2	A\G	0.11	1.13E-06	0.109	%	0.14	
PROT	6A	72549494	92.2	G\A	0.34	3.83E-07	-0.0932	%	0.21	
PROT	7A	169622110	111.9	C\A	0.07	8.50E-06	-0.114	%	0.12	
SPH	1A	52268667^E^	75.1	G\A	0.2	4.53E-06	-0.449	count	0.31	
SPH	1A	507879685	130.6	G\A	0.1	2.33E-06	0.571	count	0.20	
SPH	3B	189122341	118.9	T\A	0.07	1.18E-05	0.715	count	0.34	[79]
SPH	4A	672693794	151.3	A\C	0.22	8.87E-14	-0.830	count	0.32	
SPH	4D	217349774	81.6	T\G	0.06	3.67E-09	-1.05	count	0.68	
SPH	5B	617822981	227.0	C\A	0.22	9.20E-11	-0.917	count	0.52	
SPH	7A	672644138^A^	151.0	A\G	0.4	9.39E-09	-0.476	count	0.96	[80]
SSQM	1A	49502948^E^	73.8	T\C	0.11	4.33E-12	27.2	Spikes m^-2^	0.56	
SSQM	1A	372638734	92.9	T\C	0.07	7.38E-11	29.1	Spikes m^-2^	0.54	[73]
SSQM	2A	40249083	50.0	A\T	0.06	4.07E-07	31.3	Spikes m^-2^	0.18	
SSQM	3A	6788503	12.5	T\C	0.46	4.25E-11	15.8	Spikes m^-2^	0.35	
SSQM	6A	466270386^C^	112.9	G\A	0.34	9.45E-06	-11.9	Spikes m^-2^	0.29	
SSQM	7D	216134381	148.1	A\C	0.07	2.90E-06	-20.1	Spikes m^-2^	0.11	
STARCH	1B	39382550	47.3	G\A	0.22	3.05E-07	-0.139	%	0.72	
STARCH	2A	620961503	93.1	G\A	0.05	7.61E-06	-0.188	%	0.25	
STARCH	2D	633668158	151.2	G\T	0.07	7.23E-07	0.177	%	0.22	
STARCH	4A	122852500	59.0	A\G	0.05	1.43E-07	-0.241	%	0.31	
STARCH	4A	581248758	94.3	G\A	0.05	9.61E-09	-0.315	%	0.31	
STARCH	7A	196456932	113.6	T\G	0.05	6.42E-06	-0.185	%	0.59	
TKW	2A	740238876	138.6	C\T	0.37	1.25E-05	0.513	grams	0.25	[81–84]
TKW	2B	44983849	79.0	A\G	0.38	2.97E-07	-0.770	grams	0.12	[28,77,84,85]
TKW	4A	379041624	64.6	A\C	0.06	1.23E-05	-1.17	grams	0.44	[85,86]
TKW	6A	617591341	215.2	G\T	0.21	1.43E-08	0.703	grams	0.84	
TKW	6B	687528310	133.5	C\G	0.48	3.45E-06	-0.562	grams	0.36	
TKW	6D	467652883	184.8	C\T	0.33	1.19E-07	0.773	grams	0.95	
TKW	7A	672856129^A^	151.2	C\T	0.46	1.83E-07	-0.542	grams	0.36	[80]
TKW	7B	444463299^B^	82.4	A\G	0.06	5.85E-07	1.46	grams	0.51	[28]
TKW	7D	197300822	147.6	G\T	0.09	1.33E-06	0.772	grams	0.33	[28]
TWT	1A	583992305	218.8	C\T	0.23	1.69E-08	-2.67	g L^-1^	0.89	
TWT	3A	477707675	104.2	A\G	0.05	3.65E-08	-5.40	g L^-1^	0.56	
TWT	6A	614373502	208.5	A\G	0.34	6.58E-12	-3.14	g L^-1^	0.99	
TWT	6B	12668423	8.5	G\A	0.34	2.81E-08	2.77	g L^-1^	0.42	
YLD	2B	4638412	2.2	G\A	0.08	9.69E-06	118	kg ha^-1^	0.18	
YLD	6A	355929298	111.9	G\C	0.08	1.36E-06	-110	kg ha^-1^	0.38	
YLD	6B	656280645	110.7	C\T	0.14	9.23E-06	-75.7	kg ha^-1^	0.50	[80]
YLD	7A	30243496	32.8	C\T	0.28	2.96E-07	-66.9	kg ha^-1^	0.29	[81,84]
YLD	7D	66984783	93.8	C\A	0.31	6.41E-08	67.4	kg ha^-1^	0.71	

Chr chromosome; Pos physical position; cM genetic position in centiMorgans; Alleles major allele listed first, minor allele second, favorable allele (if any) highlighted in bold; MAF minor allele frequency; Effect mean difference in trait value caused by substituting minor allele for major allele; RMIP resampling model inclusion probability

^**a**^ FLSG flag leaf stay green; GSQM grains per square meter; GW grain weight; HD heading date; HT plant height; MAT physiological maturity date; NDVI normalized-difference vegetation index at Zadok’s GS25; PROT whole-grain protein content; SPH seeds per head; SSQM spikes per square meter; STARCH whole-grain starch content; TKW thousand kernel weight; TWT test weight; YLD grain yield

^**b**^ Superscript letters indicate QTLs with pleiotropic effects

^**c**^ Genetic position estimated from synthetic W7894 × Opata M85 GBS genetic map aligned to Chinese Spring IWGSC RefSeq v1.0 wheat reference sequence

**Fig 5:**
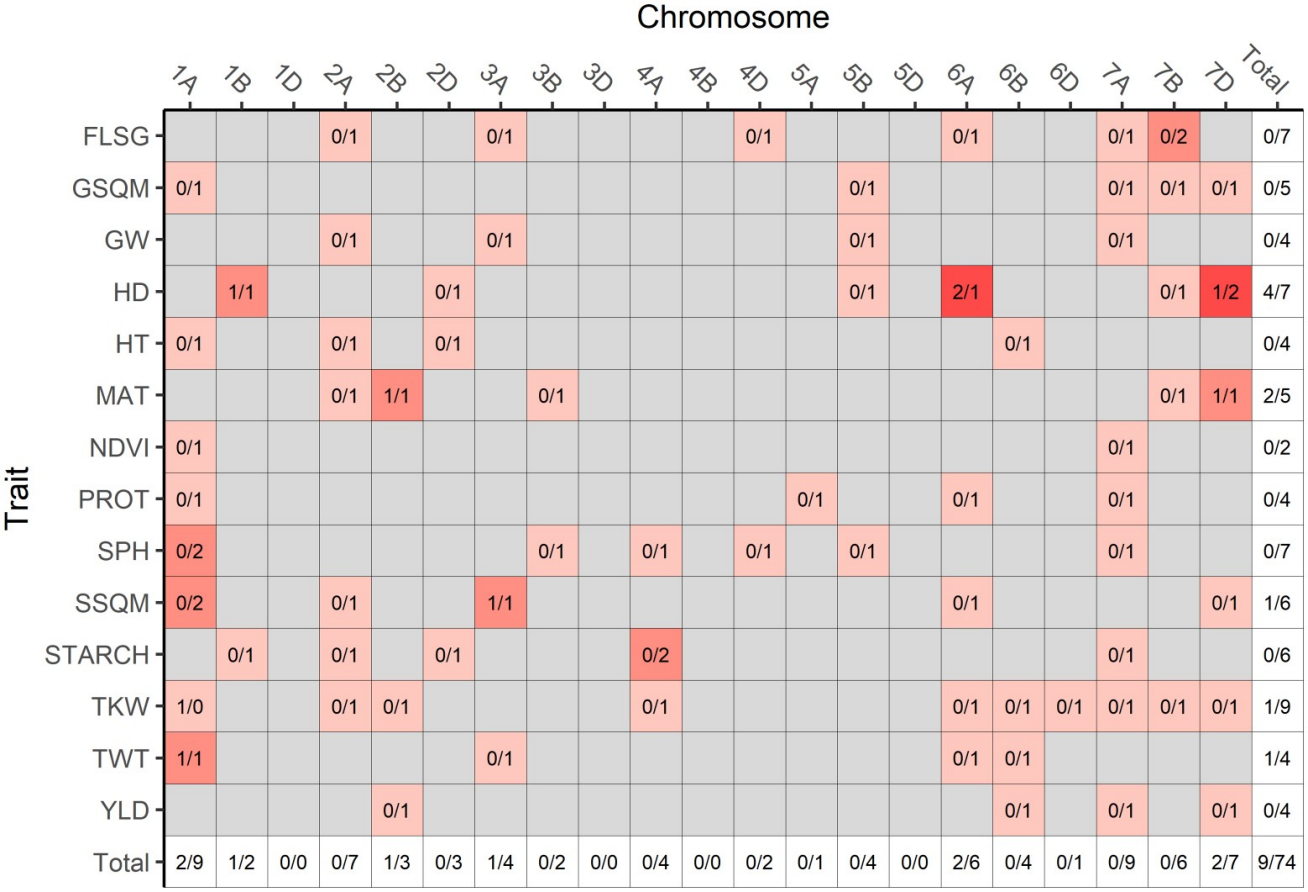
Distribution of MTAs across traits and chromosomes. The number of MTAs detected by the GCTA single-locus model (preceding slashes) and by the FarmCPU model (following slashes) are summed for each trait/chromosome combination. Grey shading indicates that no MTAs were found for that particular trait/chromosome combination. Red shading is proportional to the total number of MTAs detected by both models. Marginal totals of MTAs are displayed for each trait and chromosome, with the grand total displayed in the lower right cell.

MTAs identified by GCTA and FarmCPU were distributed sparsely among traits and chromosomes (Fig 5). The 1D, 3D, 4B, and 5D chromosomes did not contain significant MTAs for any trait. Chromosomes with high numbers of identified MTAs included: 1A, with two and nine MTAs identified by GCTA and FarmCPU, respectively; 6A, with two and six MTAs identified by GCTA and FarmCPU; 7A, with nine MTAs identified by FarmCPU; and 7D, with two and seven MTAs identified by GCTA and FarmCPU. Traits with high numbers of MTAs identified included: FLSG, with seven MTAs identified by FarmCPU; HD, with four and seven MTAs identified by GCTA and FarmCPU respectively; and TKW, with one and nine MTAs identified by GCTA and FarmCPU. The trait with the lowest number of identified MTAs was NDVI, with two MTAs identified by FarmCPU. We attempted to compare our results against those of recent GWAS studies performed in wheat (references column in Tables 3 and 4). However, the lack of a reference genome in previous studies means that QTLs can only be compared based upon genetic positions. The use of multiple marker types and genetic maps across studies makes comparisons of QTLs difficult; while we were able to compare results based on approximate genetic positions, the evidence of prior discovery in Tables 3 and 4 should be regarded as tenuous. Nevertheless, after surveying past GWAS studies of yield, yield components, and phenological traits, we categorized 4 (44%) of the MTAs detected by GCTA and 20 (26%) of the MTAs detected by FarmCPU as previously-discovered. The traits with the most previously-identified MTAs were HD, MAT, HT, and TKW; the individual MTA that was most consistently identified in prior studies was located at 44,983,849bp (79.0cM) on chromosome 2B, affecting the trait TKW.

### MTA haplotypes and translation effects

The GCTA and FarmCPU analyses identified a total of 77 significant MTAs, 35 of which were located on haplotype blocks containing two or more SNPs (S4A Table). One haplotype block spanning a 2Mb segment on chromosome 7A contained three significant MTAs located at 672,644,138bp, 672,856,129bp, and 673,460,466bp. The set of SNPs involved in MTAs overlapped 48 genes encoding 68 transcripts, and produced a total of 109 predicted transcriptional consequences due to alternative splicing (S4C Table). The most severe predicted consequences were isolated and compiled for each SNP. A total of 56 of the SNPs involved in significant MTAs (73%) were intergenic, and of these intergenic SNPs, 15 were labeled as upstream or downstream proximal variants (i.e. within 5Kb of the start or end of a gene). The remaining 21 SNPs (27%) were located within genes, with 11 of these occurring in introns, one occurring in a 5’ untranslated region, two occurring in 3’ untranslated regions, and the remaining seven causing missense mutations within exons. Putative functions for genes containing SNPs causing missense substitutions include a disease resistance protein, a RNA binding protein, a ubiquitin-family protein, a chloroplast membrane translocase, an aspartyl protease family protein, a glutamate receptor, and a *Myb* transcription factor. In addition, a total of 155 wheat genes of known function were located within the empirically-derived 1Mb radius of significant LD surrounding the set of significant MTAs (S4D Table).

## Discussion

The current study demonstrates that the recent availability of the wheat reference genome will greatly facilitate reliable QTL curation and comparisons between studies. The ability to describe MTAs in terms of both physical and genetic positions removes much of the ambiguity associated with earlier studies which relied solely on genetic positions to describe QTLs. The availability of a reference genome also enables more accurate QTL and haplotype boundary delineation and comparative genomics analyses. We were able to use positional information both to identify GBS SNPs that could potentially be used as substitutes for existing KASP SNP assays, and to identify candidate genes located near significant MTAs. Although it was difficult to compare the QTLs we identified with those discovered in previous studies, we do believe that many of the QTLs we detected were previously-characterized. These QTLs mainly affected HD, MAT, HT, and TKW, perhaps reflecting the relative ease of measurement for these traits, and the very low measurement error inherent to TKW.

This study evaluated both a traditional mixed linear model and the more-recently developed FarmCPU algorithm for performing GWAS. While a thorough comparison of these two methods is beyond the scope of this article, some general recommendations will be discussed. The authors of FarmCPU suggested that it can increase the power to detect causative genotype-phenotype associations over traditional mixed linear model methods while limiting the false-discovery rate and type I error rate [21]. A subsequent study compared single-locus mixed linear models, FarmCPU and the BayesCπ Bayesian model developed by Habier et al. [87], and found that all three offered comparable power for simple traits controlled by a few QTL, with FarmCPU offering enhanced power for moderately complex traits, and BayesCπ exhibiting the highest power for complex, highly polygenic traits [88].

Based upon our experiences, we recommend exercising some caution when using FarmCPU. Our experiences support the practice of bootstrapping of results if computational resources allow; out of 108 total MTAs identified by FarmCPU, 34 (31.5%) had RMIP values below our selected threshold of 10%. Additionally, the traditional mixed linear model identified four significant MTAs which were not identified by FarmCPU. These MTAs were also not present in the FarmCPU results which were discarded due to low RMIP values, suggesting the possibility of false negatives in the set of significant MTAs returned by FarmCPU. While FarmCPU did indeed identify many more significant MTAs than the GCTA mixed linear model, its methodology is more complex and less transparent to the user, and hence we still recommend using a single-locus mixed linear model, or perhaps a simpler multi-locus mixed model [18] to compare results.

In the present study, many previously-characterized loci of agronomic importance interrogated with KASP-SNP assays had significant effects on multiple traits (S3B Table). Loci that significantly affected many traits included the *Sr36* stem rust resistance gene and the *TaSus2-2B* sucrose synthase gene (both assaying the presence/absence of the 2G:2B *T*. *timopheevi* translocation), the 1AL:1RS and 1BL:1RS translocations, the *Ppd-B1* and *Ppd-D1* photosensitivity genes, the *Rht-B1* and *Rht-D1* dwarfing genes, and polymorphisms within the *Vrn-A1* vernalization gene. However, despite their significant effects on many traits, the KASP assays for these loci were not among the significant MTAs identified in the current study. Some potential explanations for these results are presented below.

The two *Rht* dwarfing genes *Rht-B1b* and *Rht-D1b* mostly occurred in repulsion within the testing panel. Of the 322 lines included in the panel, 22 had neither the *Rht-B1b* nor the *Rht-D1b* dwarfing alleles, one line was heterozygous for both alleles, and one line was homozygous for both alleles, with the rest being homozygous for either one dwarfing allele or the other. Note that lines lacking both *Rht-B1b* and *Rht-D1b* may contain other dwarfing genes which were not assayed in this study. At face value, this high degree of repulsion led to the somewhat odd finding that the presence of the *Rht-B1b* allele increased height and decreased yield when allelic effects were calculated for each of the KASP assays individually. A subsequent ANOVA pooling together the two dwarfing alleles revealed that at an alpha level of 0.05, lines with either *Rht-B1b* or *Rht-D1b* were significantly shorter and yielded significantly higher than lines that were wild-type for both genes. Yield was significantly higher for lines with only *Rht-D1b* vs. those with only *Rht-B1b*, though height was not significantly different between lines with these dwarfing genes. (These analyses excluded lines that were heterozygous for either allele, and the single line that was homozygous for both alleles). This perhaps serves as a good reminder of how population-specific epistasis can prevent the accurate estimation of many allele effects. While multi-locus GWAS models may partially ameliorate these problems in some cases, the careful choice of experimental design, such as the use of nested association mapping panels [89], is likely a better solution.

In the present study, both the *Sr36* and *TaSus2-2B* genes were highly confounded with the testing panel’s population structure. A SNP located at 44,983,849bp on chromosome 2B was in moderate LD with both *Sr36* and *TaSus2-2B* (r^2^ = 0.24), and had the expected significant impact on TKW, given the known effects that *TaSus2-2B* exerts on kernel weight [13]. The 2G:2B translocation from *T*. *timopheevi* on which the *Sr36* gene resides introduced a large alien haplotype into the panel germplasm. Cavanagh et al. [90] found that *Sr36* was associated with a large segment of segregation distortion on chromosome 2B in seven different mapping populations. In the present study, splitting the population by the presence or absence of the *Sr36* gene and subsequently performing a genome-wide F_ST_ scan (Fig 3) revealed a large high-LD block spanning most of chromosome 2B, containing many SNPs which could almost perfectly differentiate between lines with and without the 2G:2B translocation. In addition, an enrichment in high-F_ST_ SNPs was observed on chromosomes 2A and 2D, likely due to SNP misalignments between chromosomes within this homeologous group. On chromosome 2B, there was a general positive linear trend between a SNP’s *F*_*ST*_ score and its LD with the *Sr36* KASP marker. This was as expected, as the presence or absence of this marker was used to partition the subpopulations. However, a smaller subset of SNPs simultaneously exhibited high F_ST_ and an r^2^ value with the KASP *Sr36* assay that approached zero. These SNPs were distributed throughout the translocation (Fig 4). It is likely that SNPs with a high F_ST_ value that are also in high LD with the *Sr36* KASP marker share a common private haplotype allele inherited from *T*. *timopheevi*, while those with a high *F*_*ST*_ are in low LD with the *Sr36* KASP assay represent a set of SNPs with shared alleles between *T*. *aestivum* and *T*. *timopheevi*, where recombination rates are higher. Despite the effects of the 2G:2B translocation in the current study, population structure was generally subdued, as evidenced by principal component plots of the LD-thinned dataset, where the first principal component explained only 3.68% of total variation (Fig 1B). This finding is in line with those of previous studies examining population structure in elite European winter wheat germplasm [91,92]. This suggests extensive past admixture among the lines included in the population, which is as expected given the frequent germplasm exchanges that are typical of public small grains breeding programs.

A previous report by Sukumaran et al. [31] found much more pronounced population structure effects due to rye translocations, using a panel of elite spring germplasm which clustered into two distinct sub-populations explained by the presence or absence of the 1BL:1RS translocation. In addition, Sukumaran et al. found that the 1BL:1RS translocation explained significant differences among the two subpopulations for most of the traits included in their study (e.g. grain yield, grain number, grain weight, plant height, and several phenological traits). The 1AL:1RS and 1BL:1RS translocations have been associated with desirable disease and insect resistance traits, as well as drought and general environmental stress resistance. However, the 1BL:1RS translocation has been associated with lateness, and effects on yield due to these translocations may be manifested depending upon environment and genetic background [93–95]. While both the 1AL:1RS and 1BL:1RS translocations did produce significant differences for many traits in this study (S3B Table), their contributions to population structure were not noticeable in comparison to the effects of the 2G:2B translocation (data not shown). In addition, the indicator KASP markers for these translocations did not produce significant p-values in either the GCTA or FarmCPU GWAS. The 1AL:1RS translocation occurred at a very low frequency (0.07) within the tested germplasm, while the 1BL:1RS translocation occurred at a frequency of 0.19. In the study of Sukumaran et al. [31], the 1BL:1RS translocation occurred at a frequency of 0.39. In the present study, the KASP markers for 1AL:1RS and 1BL:1RS were in high LD (r^2^ approximately 0.8) with the SNPs located at 103,223,278bp on chromosome 1A and 39,382,550bp on chromosome 1B respectively. This high LD suggests that these GBS markers, or others in close proximity, could be used as suitable indicators for the 1AL:1RS and 1BL:1RS translocations. Although further validation is required, ideally GBS markers, individually or in groups, could be used to ascertain the presence or absence of these translocations without necessitating the use of the additional standalone assays that are currently performed.

The effects of previously-characterized genes affecting phenological traits were likewise insignificant in the GWAS, likely due to a variety of factors. Polymorphisms of *Vrn-A1* exon 7 and *Vrn-B1* occurred at low frequencies of 0.03 and 0.07 within the testing panel, respectively, most likely precluding their detection in the GWAS. The *Vrn-A1* exon 4 SNP had a higher minor allele frequency of 0.14, and was in moderate LD (r^2^ = 0.29) with one GBS SNP located at 594,957,276bp on chromosome 5A. The *Vrn-A1* KASP assays used in this study may simply have not been predictive enough to adequately distinguish among different vernalization alleles present at the *Vrn-A1* locus. The *Ppd* loci were likewise never identified as significant, most likely due to masking from the predominance of long-vernalizing genotypes in the panel. However, a BLAST analysis indicated that the GBS SNP located at 35,084,672bp on chromosome 2D, which FarmCPU identified as significant for the trait HD, was located within approximately 1Mb of *Ppd-D1*.

As previously mentioned, there was evidence for pleiotropic effects involving multiple SNPs and traits in both the GCTA and FarmCPU results (Tables 3 and 4). The sole region of pleiotropic effect identified by GCTA was a QTL located at approximately 58.5Mb (84.6 cM) on chromosome 7D, affecting the phenological traits HD and MAT. The haplotype block analysis indicated that this QTL spanned a region of roughly 1.5Mb. This QTL was also identified by FarmCPU, and was one of the more consistently significant QTLs identified, with a RMIP value of 0.78. Many of the other pleiotropic QTLs identified by FarmCPU demonstrated trait compensation effects relating to grain number and grain weight. For example, the minor allele of a QTL located at approximately 444Mb (82.4cM) on chromosome 7B produced an increase in TKW and a decrease in GSQM, while the minor allele of a QTL at approximately 50Mb (75cM) on chromosome 1A produced an increase in SSQM, but a slight decrease in SPH. The minor allele of a QTL on chromosome 7A at approximately 673Mb (151cM) caused a decrease in TKW, and an increase in GSQM. It also apparently produced slight decrease in SPH, though the effects of this locus will be discussed in greater detail below. Finally, a large region on chromosome 6A affected the relatively uncorrelated traits HD and SSQM. It is not clear whether this region is being affected by long-range LD, or simply physical linkage, as the distance between the two MTAs involved is approximately 16.5Mb. A simple pairwise LD analysis indicated that these SNPs were in moderate LD with each other, though the haplotype block analysis placed them in separate haplotypes.

Due to the large number of significant findings, we limit further discussion of the GWAS results to several MTAs in close proximity to plausible candidate genes (S5 Table). The previously-mentioned pleiotropic region on chromosome 7A affecting the traits TKW, SPH, and GSQM spanned a distance of approximately 1.5Mb, containing three MTAs. FarmCPU identified one significant SNP within this haplotype with a RMIP value of 0.96. The 7A pleiotropic region contains a total of 22 genes, only three of which have listed functions in UniProt. However, the IWGSC’s automated gene functional annotation process [34,96] had previously identified one gene within this haplotype as a likely ortholog of the aberrant panicle organization 1 (*APO1*) protein, which plays an important role in panicle architecture and development in rice (*Oryza sativa* L.). The loss of function of *APO1* results in rice plants which produce small panicles with a lower number of inflorescence branches and spikelets [97]. The minor alleles for two of the MTAs in this region decreased TKW and increased GSQM. We also found that the minor allele of the third MTA in this region decreased SPH, which was against expectation. However, the high MAF of all three MTAs within this QTL (0.4 and 0.46) suggested the possibility that at least one was actually in *trans* linkage with the other two. Upon closer inspection of the SNP data, we discovered that this was indeed the case, with the SNP associated with SPH in *trans* configuration with the others. Therefore, we detected two primary haplotypes present at this QTL with frequencies close to 0.5: one increasing GSQM and SPH while decreasing TKW, and the other with the opposite effect. The MTAs for SPH and TKW are not in perfect LD; a total of 40 lines (12.4%) exhibited recombination between these SNPs. The SNP associated with TKW within the 7A QTL is predicted to cause a missense protein translation effect within an aspartyl protease family protein, though it is not known whether this is relevant to the QTL’s phenotypic effects. The fact that FarmCPU fit different covariates for different traits within this region raises the possibility that this QTL’s effects could be due to multiple candidate genes, rather than a single gene such as *APO1* exhibiting pleiotropic effects. However, we cannot at this time test this hypothesis.

Chromosome 7B also contained a large number of putative candidate genes for multiple traits. A pectin esterase was located in close proximity (67Kb) with a MTA affecting FLSG located at 64Mb (62.4cM). Due to the ubiquity of pectin within plant cell walls, pectin esterases have been associated with a wide range of cellular processes (reviewed in [98]). Notably, pectin esterases have been found to play a role in fruit maturation in heat-stressed tomato plants [99], as well as the initiation of flowering in day lilies [100]. Another MTA on 7B located at 97Mb (65.4cM), affecting the trait HD, was within 485Kb of a MYB transcription factor. The MYB family of proteins function in a wide number of signal transduction pathways in plants. Gibberellin-interacting MYB factors (GAMYBs) were first identified in barley (*Hordeum vulgare* L.), inducing the activation of alpha-amylase in the aleurone tissue of grains [101]. GAMYB factors were subsequently identified in wheat [102]. Currently, GAMYB factors have been putatively implicated in contributing to flowering initiation, though their role in this process is not well understood. Gocal et al. [103] suggested that a GAMYB protein in the grass species *Lolium temulentum* L. was an important component in signaling pathways for flower development. However, Kaneko et al. [104] later found that while rice GAMYBs were important in normal flower organ development, knockout mutants did not display any differences from wild-type plants in the timing of flower development. Therefore, while GAMYB factors may play a role in wheat floral development, their exact function in this process remains unknown. A pleiotropic SNP located at approximately 444Mb (82.4cM) on chromosome 7B exerted large effects on the traits GSQM and TKW, and was intronic within an ortholog of an intracellular protein transporter identified in *Medicago truncatula* Gaertn. While this SNP is not associated with a haplotype block, it is located 170Kb from a glycosyltransferase gene. The glycosyltransferases are a superfamily consisting of thousands of identified proteins. Notably, multiple families of glycosyltransferases have been associated with grain development in wheat [105].

On chromosome 7D, a SNP located at approximately 59Mb (84.6cM) was identified by both the single-locus and FarmCPU models, produced pleiotropic effects for both HD and MAT, had a large effect size magnitude of approximately 0.5 days, and produced a RMIP value of 0.78 for HD. Due to its exertion of effects with the same sign on both HD and MAT, it is likely that this locus could be involved with plant vernalization or photoperiod response. While this SNP is approximately 10Mb away from the *VRN3* vernalization response/flowering time gene, a significant MTA located at 66,984,783bp on 7D affecting yield is located only 1.5Mb away from *VRN3*, and there is no significant LD between these two SNPs, making it likely that some other as yet undetermined causal agent is underlying this locus’ effects on heading and maturation dates. This SNP is intronic within an ortholog of the *Escherichia coli* Acetyl-coenzyme A carboxylase carboxyl transferase subunit alpha gene, which is involved in fatty acid synthesis via the synthesis of malonyl-CoA [106], though any connection between this function and vernalization response in plants remains unknown.

Several other MTAs were in close proximity to candidate genes that have previously been functionally characterized in wheat. One MTA on chromosome 1A affecting the trait GSQM was located 409Kb away from the gibberellin oxidase gene *TaGA2ox-A1*. Gibberellins are an important family of plant hormones, which have many functions in plant growth and developmental processes, including a crucial role in grain volume increase in wheat [107]. The GA2ox family of oxygenases play a role in deactivating gibberrelins, and paralogs of many GA2ox genes have been identified in all three genomes of hexaploid wheat [108]. On Chromosome 3A, the GCTA and FarmCPU models both detected a MTA affecting the trait SSQM, located within 506Kb of the gene *TaMFT*. This gene, an ortholog of the *Arabidopsis thaliana* gene *MFT*, encodes a protein which functions as a key promoter of seed dormancy and suppressor of precocious seed germination during seed development [109]. Finally, one MTA located on chromosome 1A, affecting the trait SPH, is located within 76Kb of *TaB2*, a wheat ortholog of a protein first isolated from carrot (*Daucus carota* L.) [110]. In wheat, B2 proteins were found to function as heat stress response proteins, and are highly upregulated in developing seeds during and for several days following the application of heat stress [111]. Additionally, *TaB2* was found to influence plant growth and development upon transformation into *Arabidopsis thaliana* [112].

Taken together, these findings suggest a viable set of candidate genes and QTLs that may be exploited to increase yield in soft winter wheat breeding. The recent use of CRISPR-Cas9 genome editing systems in wheat (e.g. [113–116]) may allow for rapidly testing the functions of the candidate genes mentioned above without the need for expensive and time-consuming fine mapping and gene cloning. The finding of multiple MTAs affecting phenological traits suggests that there is still variation in the tested elite germplasm that can be exploited in breeding programs to fine-tune the timing of growth stages and grain fill duration, perhaps allowing for finer control of maturation date to maximize grain fill time while avoiding heat stress during kernel formation. The findings regarding traits relating to grain density per unit area and grain weight give cause for hope, while also suggesting strategies for ongoing germplasm improvement. Many MTAs for these traits appeared to entail no significant tradeoff between grain number and grain size. These included MTAs for GSQM on chromosomes 1A and 5B, MTAs for SSQM on chromosomes 1A, 2A, 3A and 7D, and MTAs for TKW on chromosomes 2A, 2B, 4A, 6A, 6B, 6D and 7D. Barring any epistatic effects, favorable alleles for these QTLs could gradually be combined within the tested elite germplasm pool. In contrast, several QTLs located on chromosomes 1A, 7A, and 7B exerted pleiotropic effects for these traits, with favorable alleles in a *trans* configuration. If these QTL represent tightly *trans*-linked genes, then they may eventually be exploited if the component genes are broken up by recombination over generations. However, QTLs that represent true pleiotropic effects of a single gene will likely not be useful for achieving genetic gain. Many of the MTAs for GSQM, TKW, and particularly SSQM had favorable minor alleles, suggesting either that breeders have unintentionally performed selections which favored the less-desirable allele, or that these favorable alleles initially occurred at low frequencies and remain rare due to a lack of selection pressure.

While the availability of a reference genome allows for previously impossible follow-up analyses, this study also identifies a number of areas for continued improvement for GWAS experiments in wheat. For instance, the genome-wide *F*_*ST*_ scan based upon the presence or absence of *Sr36* revealed multiple SNPs which were misaligned between the group 2 homeologous chromosomes, and therefore non-allelic. In addition to the obvious problem of potentially identifying significant MTAs on the wrong chromosome, these SNPs complicate the process of haplotype estimation, as they may artificially “break” patterns of strong LD within haplotype blocks. There is currently no simple solution to identify misaligned SNPs within GBS datasets, as discarding sequencing reads that don’t uniquely map to a single location may entail discarding a large portion of data. However, several methods for identifying non-allelic SNPs in polyploids are under development (e.g. [117,118]). Finally, although the availability of a reference genome has greatly aided the interpretation of GWAS studies in wheat, it should be noted that the causal variants underlying the majority of significant MTAs identified in this study remain unknown. As the amount of bioinformatics data available for wheat increases, this situation may improve through the increasing availability of gene expression data and gene ontology information, enabling the use of new techniques such as gene set analysis.

## Conclusions

The significant MTAs reported in this study indicate that there is still genetic variation in the tested elite germplasm that may be exploited for yield gains. In particular, the combination of identified MTAs affecting traits relating to grains per unit area and phenological development offer promise for increasing the former while avoiding the penalizing effect of lower average grain weights. In addition, this study suggests that GBS markers can be used to capture much of the variance explained by previously-characterized polymorphisms of major effect. We made use of the first reference genome assembled for wheat, enabling the identification of MTAs based on both physical and genetic positions; it is hoped that the ability to anchor MTAs by physical position will lead to better curation of results and consistency across GWAS studies in the future. This study also identifies some potential targets for future *in vitro* studies to ascertain the biological functions of several candidate genes affecting yield-related traits in wheat. Future challenges will include the proper design of GBS or other genotyping assays to capture the effects of previously-characterized polymorphisms while simultaneously allowing for the discovery of novel polymorphisms affecting traits of interest, better identification of non-allelic SNPs which are misaligned between homeologous chromosomes, introgression of multiple favorable alleles into suitable genetic backgrounds, and more thorough characterization of gene functions to ease the identification of candidate genes following association analyses.

## Supporting information

S1 Table List of germplasm tested in the study. (XLSX)

S2 Table Description of traits examined in the study, with trait ontologies as described in http://www.planteome.org/. (XLSX)

S3 Table Design of KASP SNP assays for interrogating previously-characterized loci of major effect, and a summary of the allelic effects of these loci. (XLSX)

S4 Table List of genes overlapping significant SNPs and haplotype blocks, predicted protein translation effects for all significant SNPs, and list of all wheat genes with annotations in UniProt occurring within 1Mb of significant MTAs. (XLSX)

S5 Table Summary information for identified candidate genes. (XLSX)

S1 Fig Individual and cumulative portions of variance explained by the first 25 principal components of the imputed genotypic data, prior to LD-based filtering. (TIFF)

S2 Fig Number of SNPs per chromosome following the application of all genotypic data filtering steps. (TIFF)

S1 File Manhattan and QQ plots for SNP p-values generated by the GCTA leave-one-chromosome-out (LOCO) mixed linear model GWAS. (PDF)

S2 File Manhattan and QQ plots for SNP p-values generated by the FarmCPU GWAS. (PDF)
