# Supplementary figures and images for: Genome-wide association studies for yield-related traits in soft red winter wheat grown in Virginia
Source: PLoS One. 2019 Feb 22;14(2):e0208217. doi: 10.1371/journal.pone.0208217 (PMC6386437; doi:10.1371/journal.pone.0208217)

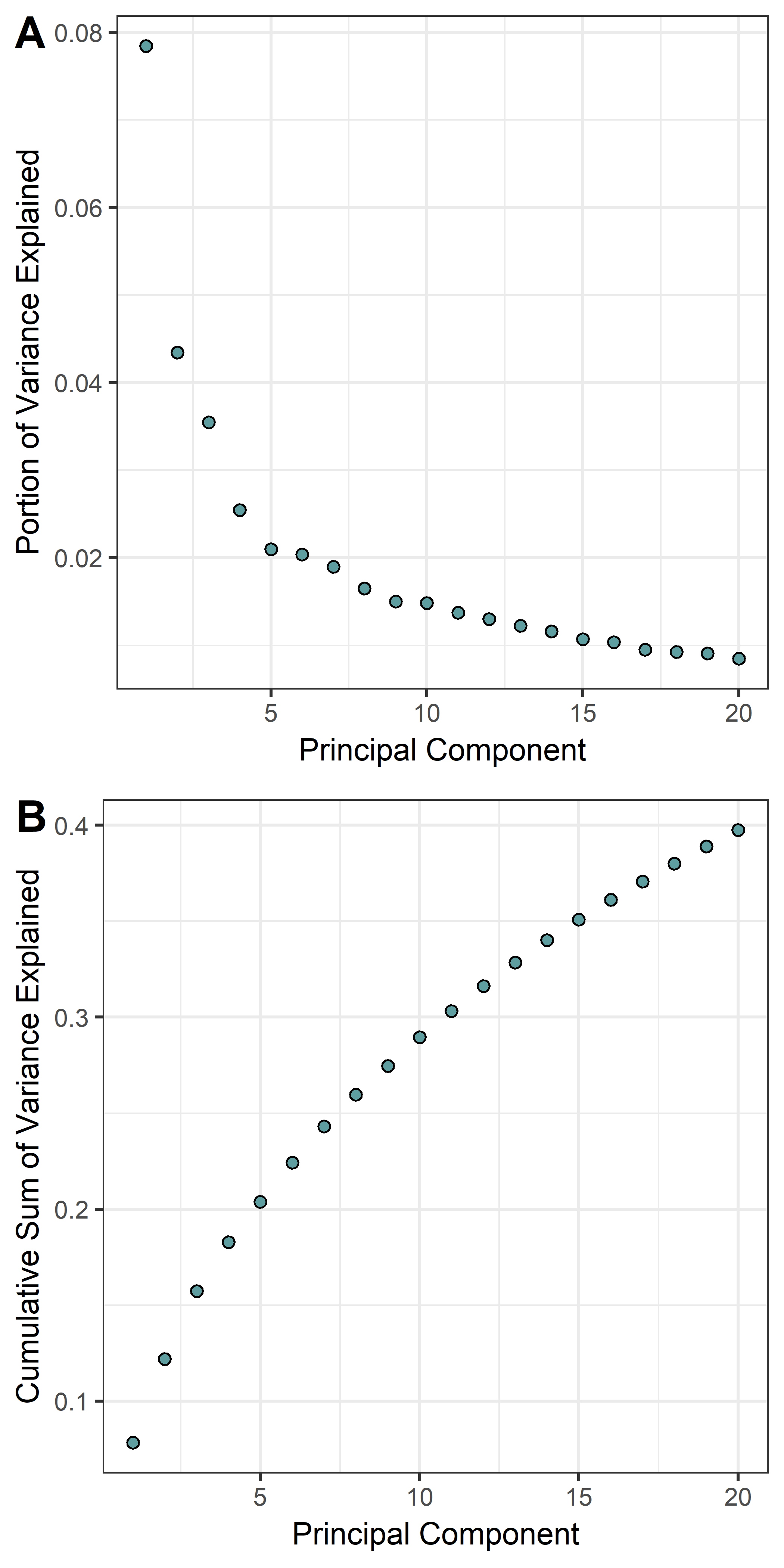

Supplement: S1 Fig — (TIFF) [file pone.0208217.s006.tiff]

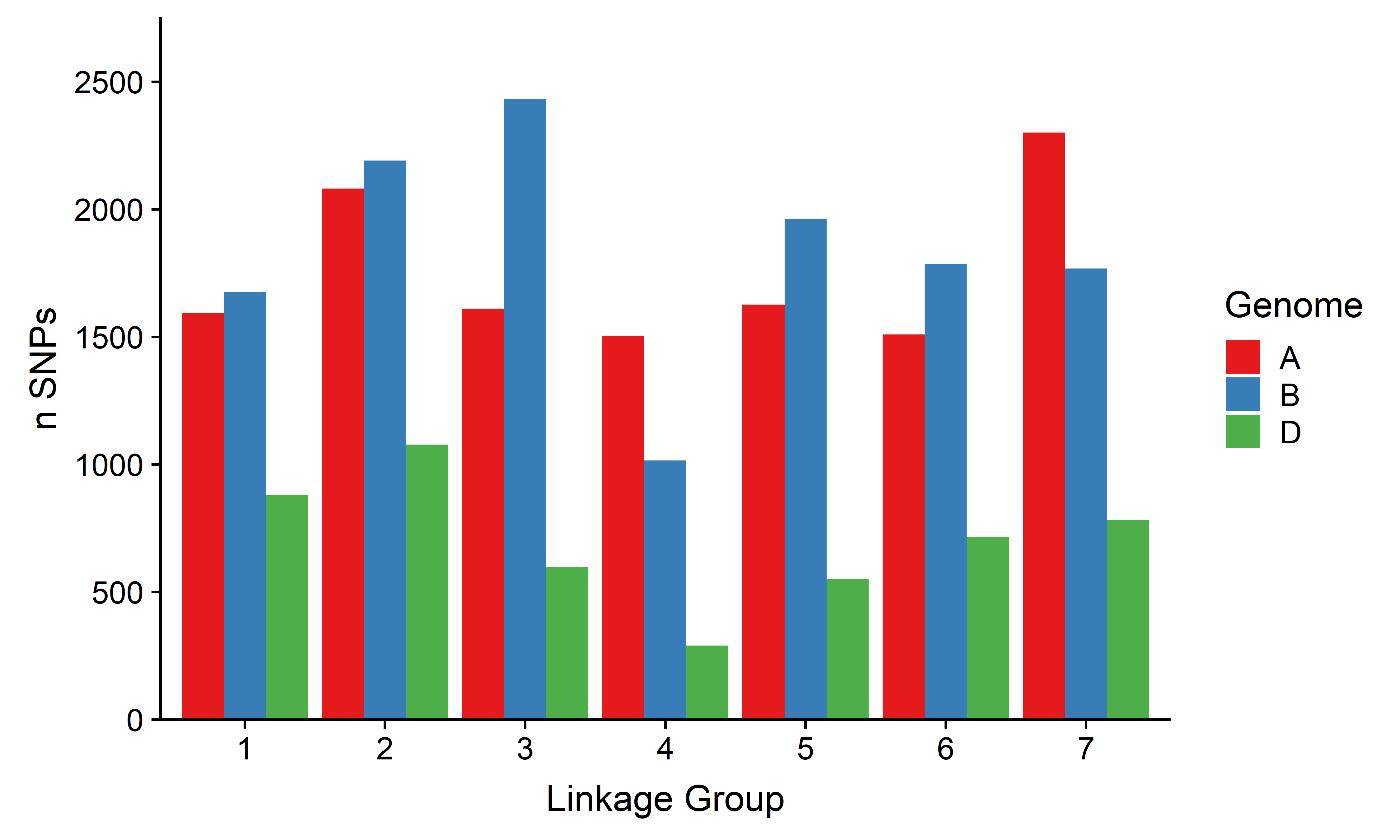

Supplement: S2 Fig — (TIFF) [file pone.0208217.s007.tiff]
