# Supplementary material for: Genome-wide association studies for yield-related traits in soft red winter wheat grown in Virginia
Source: PLoS One. 2019 Feb 22;14(2):e0208217. doi: 10.1371/journal.pone.0208217 (PMC6386437; doi:10.1371/journal.pone.0208217)

Genome-wide association studies for yield-related traits in soft red winter wheat grown in Virginia

Brian P. Ward, Frederic L. Kolb, David A. Van Sanford, Gina Brown-Guedira, Priyanka Tyagi, Clay H. Sneller, and Carl A. Griffey

S1 File. Manhattan and QQ plots for SNP p-values generated by the GCTA leave-one-chromosome-out method.

Below are Manhattan and uniform distribution quantile-quantile (QQ) plots produced from the output of a GWAS analysis performed using the leave-one-chromosome-out (LOCO) mixed linear model method in GCTA.

A total of 29,949 genotyping-by-sequencing (GBS) generated markers were included in the analysis.

A false-discovery rate (FDR) threshold was used to determine signficance for individual SNP p-values. A horizontal line is placed on the Manhattan plot only if at least one SNP's -log(p) value exceeds the FDR threshold.

Manhattan and QQ plots for trait FLSG

No SNP adjusted p-values exceed the FDR threshold of 0.05

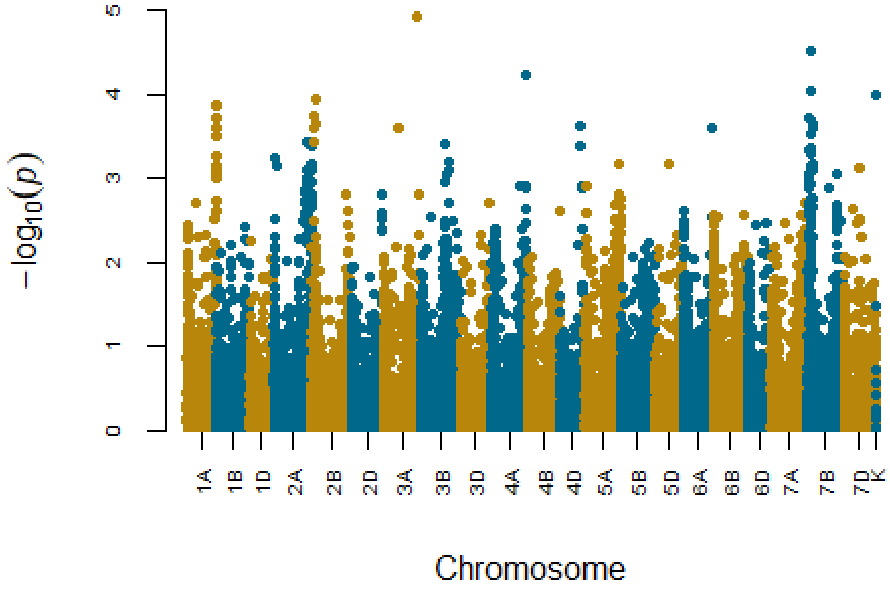

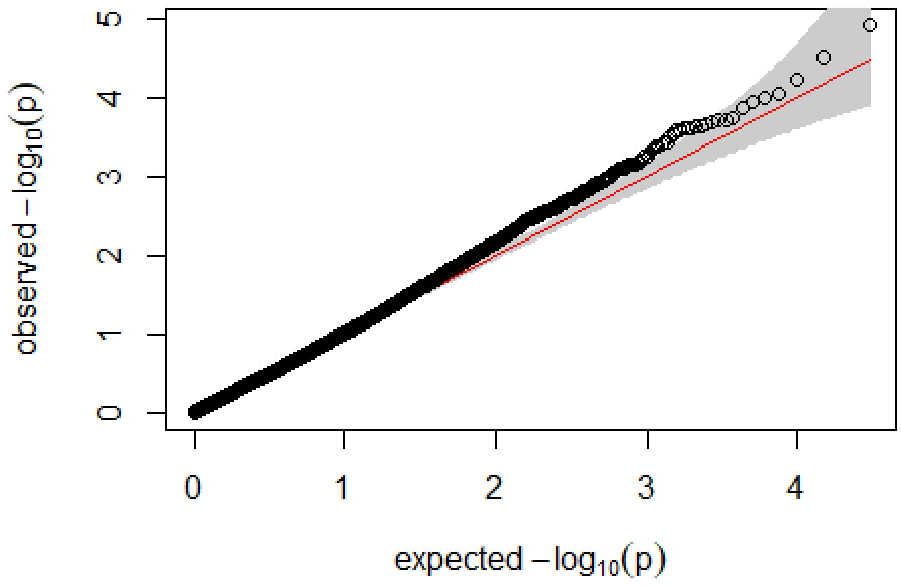

Manhattan and QQ plots for trait GSQM

No SNP adjusted p-values exceed the FDR threshold of 0.05

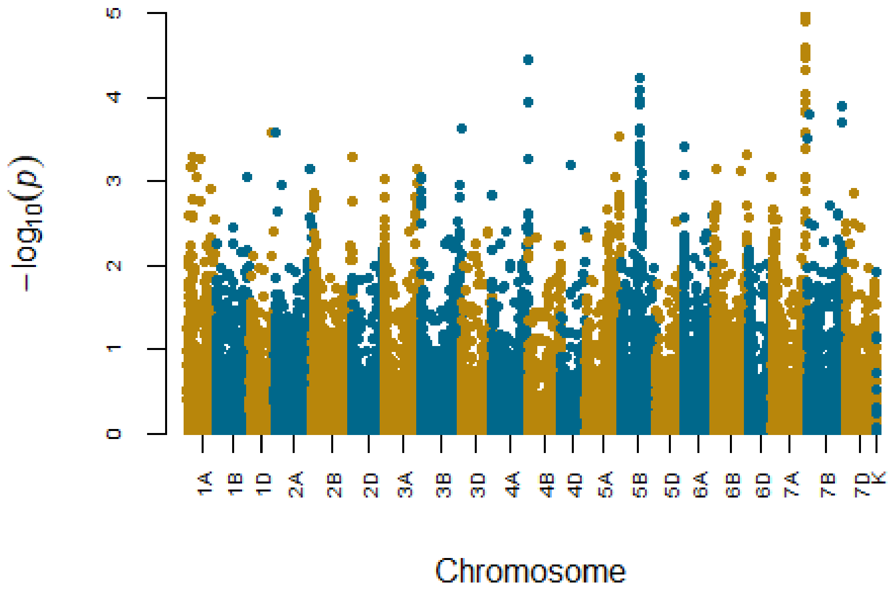

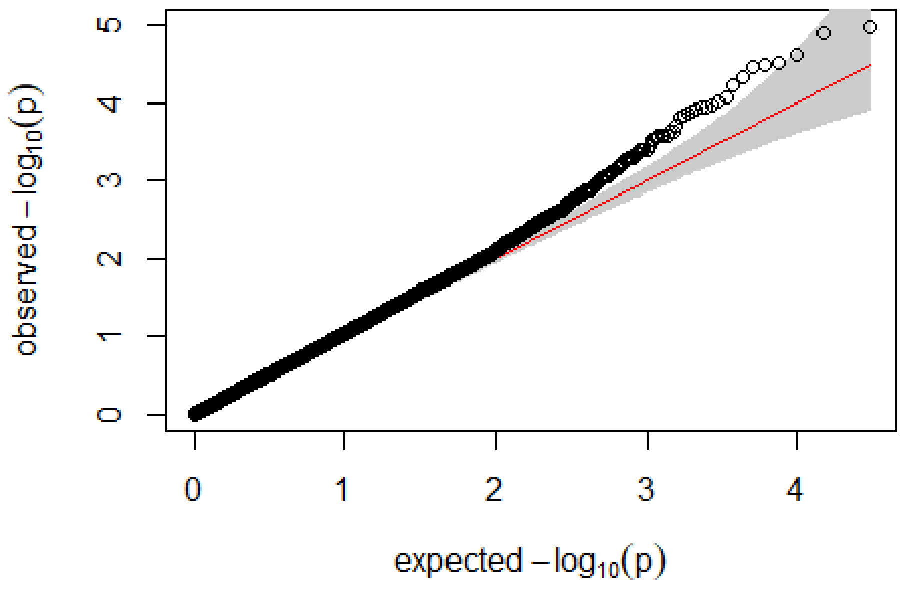

Manhattan and QQ plots for trait GW
No SNP adjusted p-values exceed the FDR threshold of 0.05

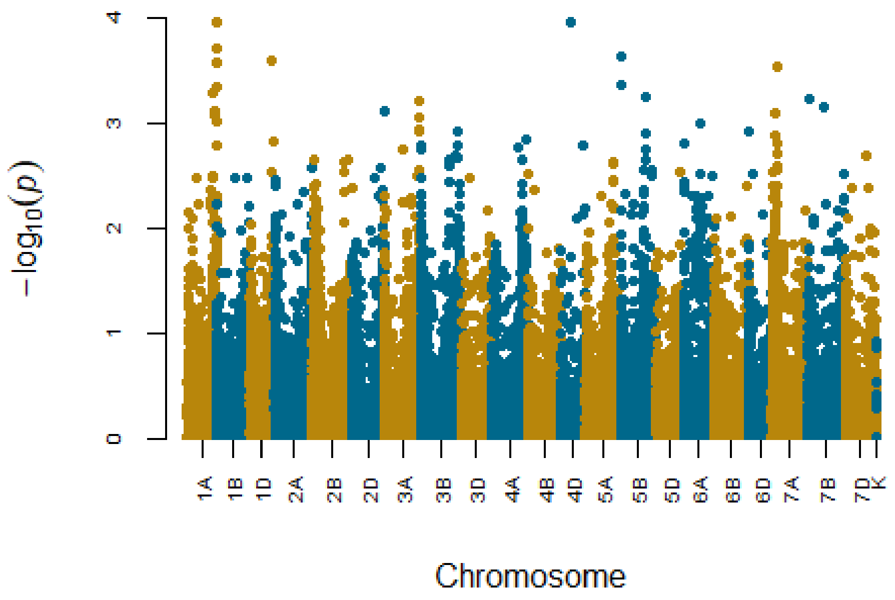

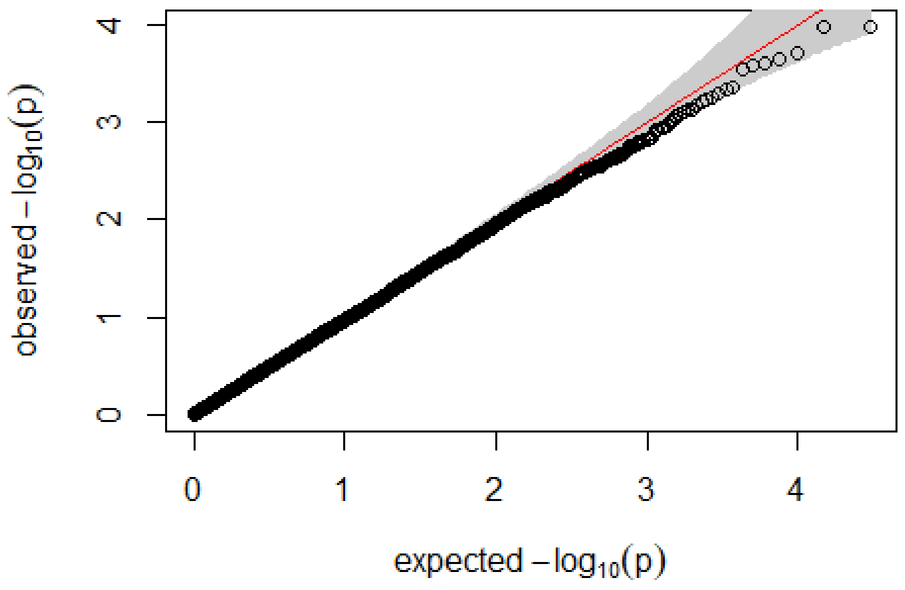

Manhattan and QQ plots for trait HD

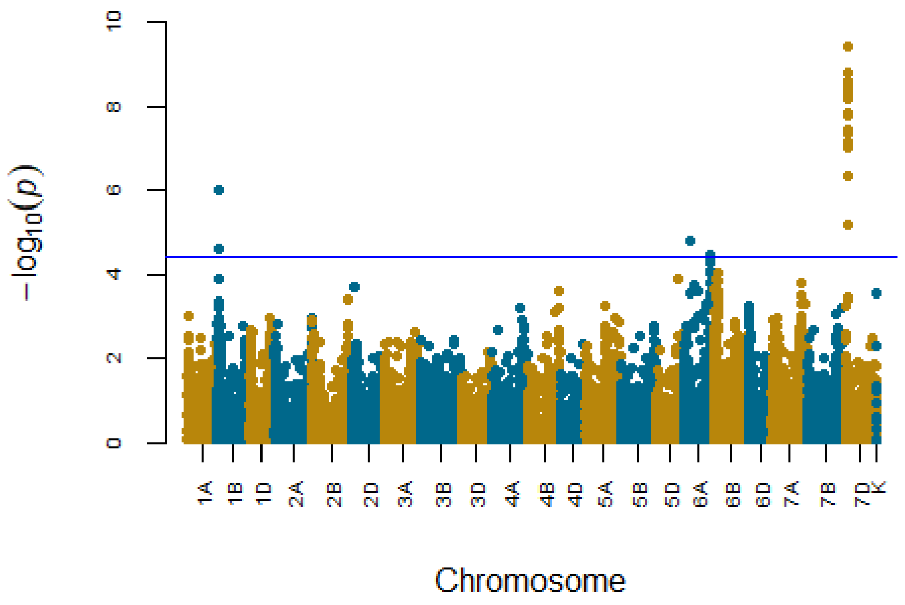

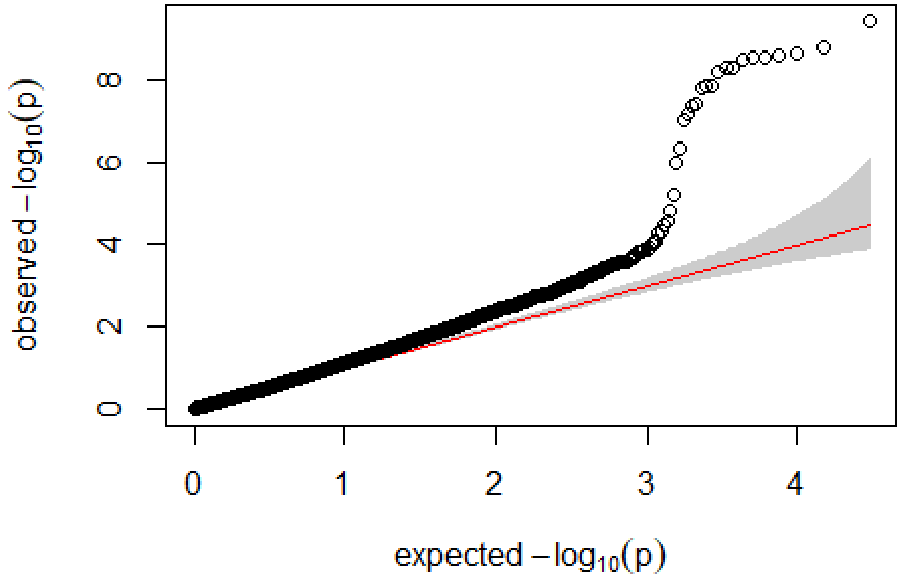

Manhattan and QQ plots for trait HT
No SNP adjusted p-values exceed the FDR threshold of 0.05

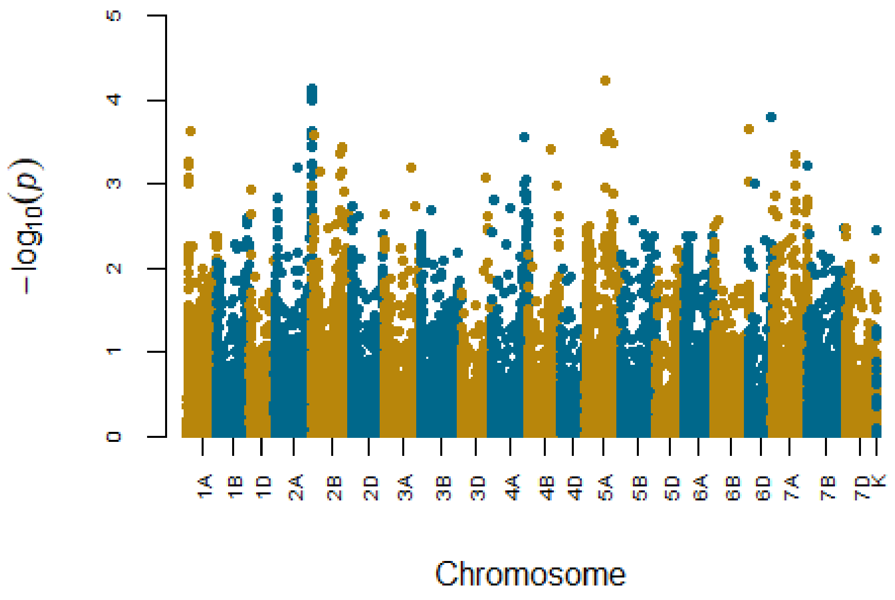

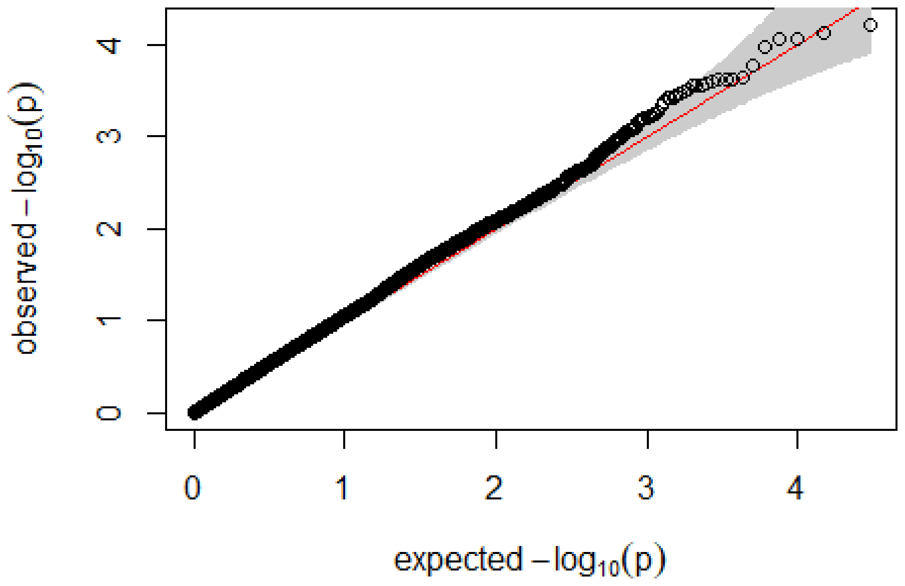

Manhattan and QQ plots for trait MAT

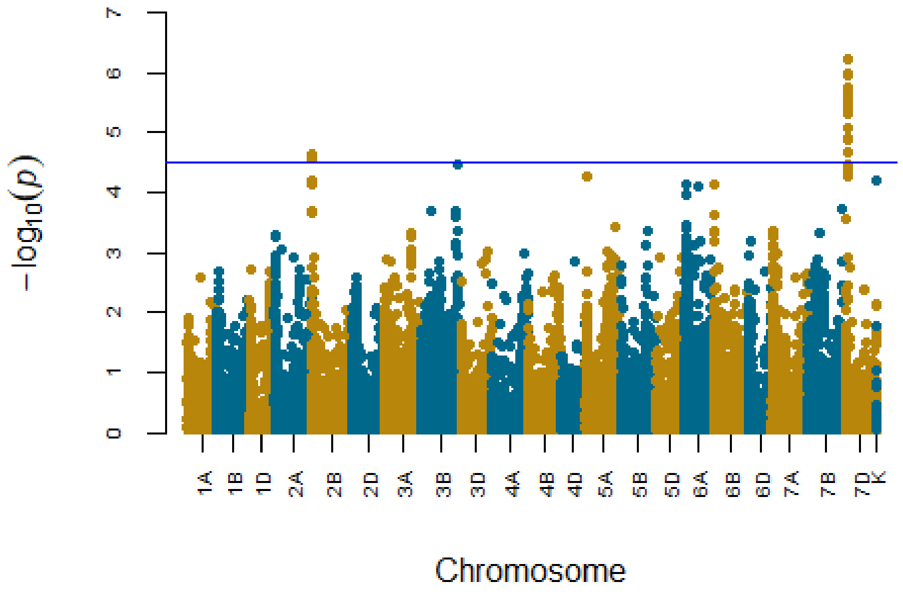

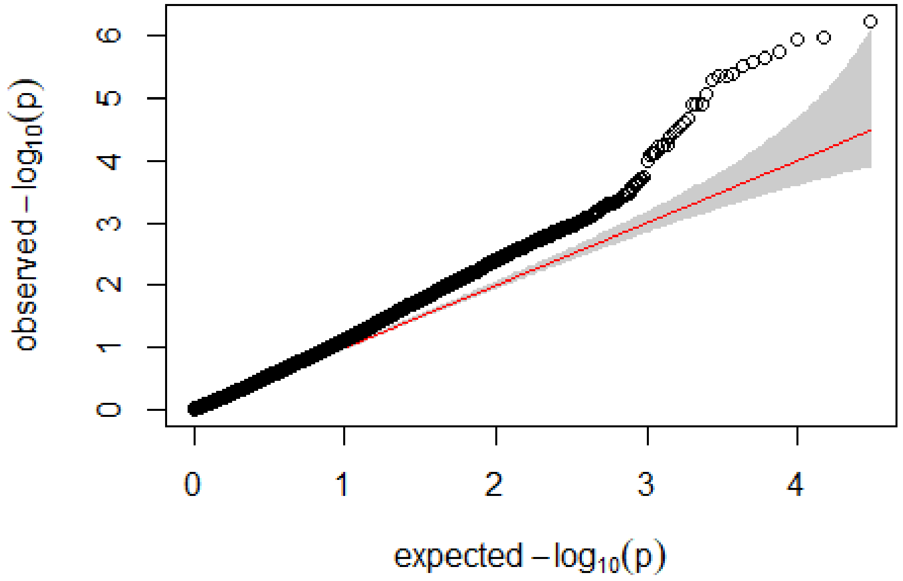

Manhattan and QQ plots for trait NDVI

No SNP adjusted p-values exceed the FDR threshold of 0.05

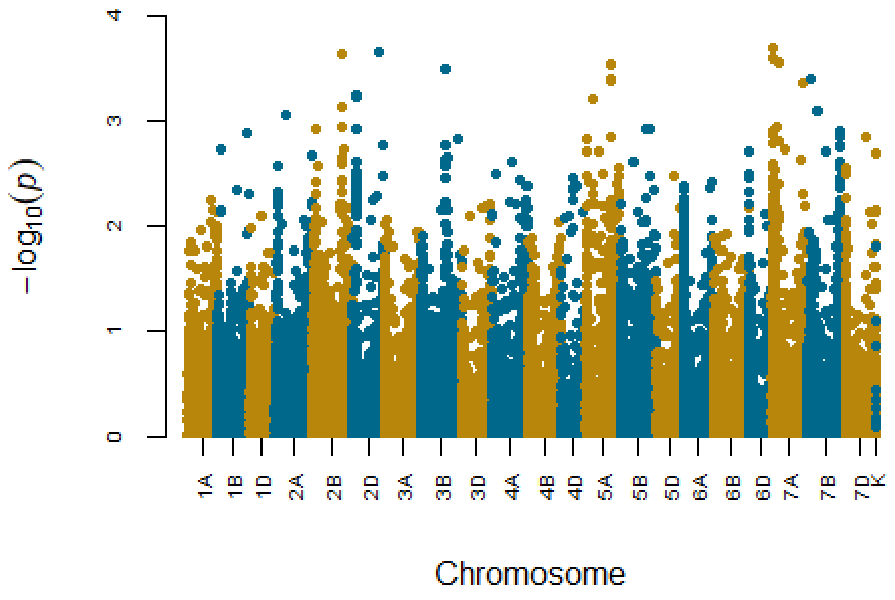

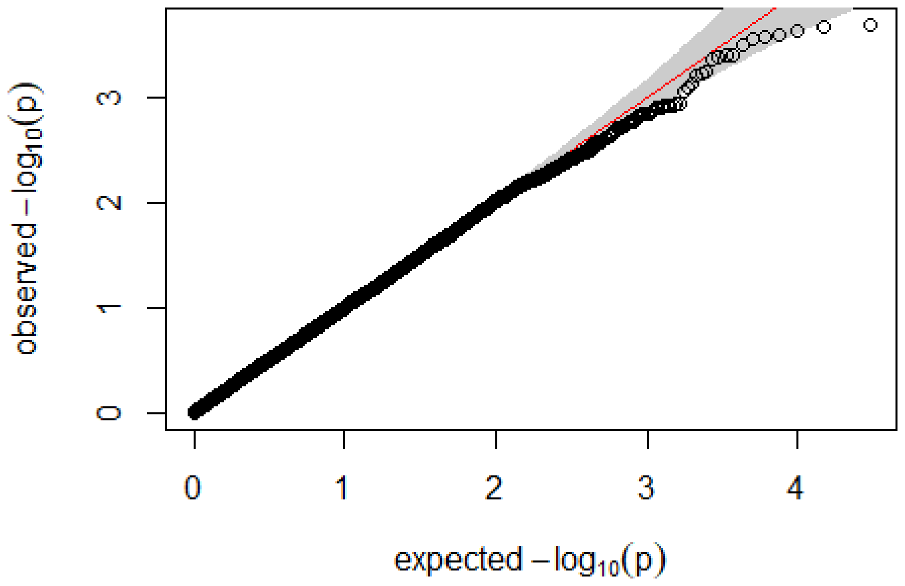

Manhattan and QQ plots for trait PROT
No SNP adjusted p-values exceed the FDR threshold of 0.05

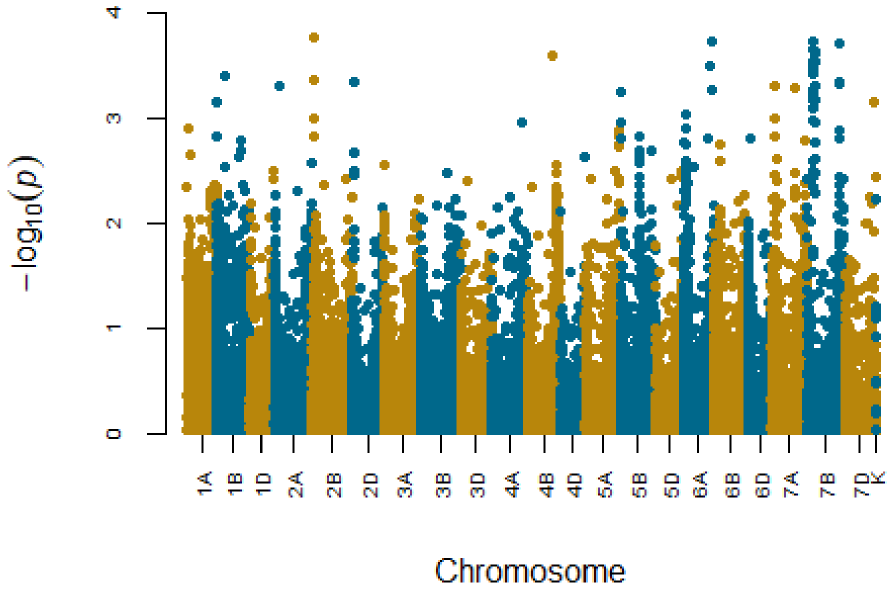

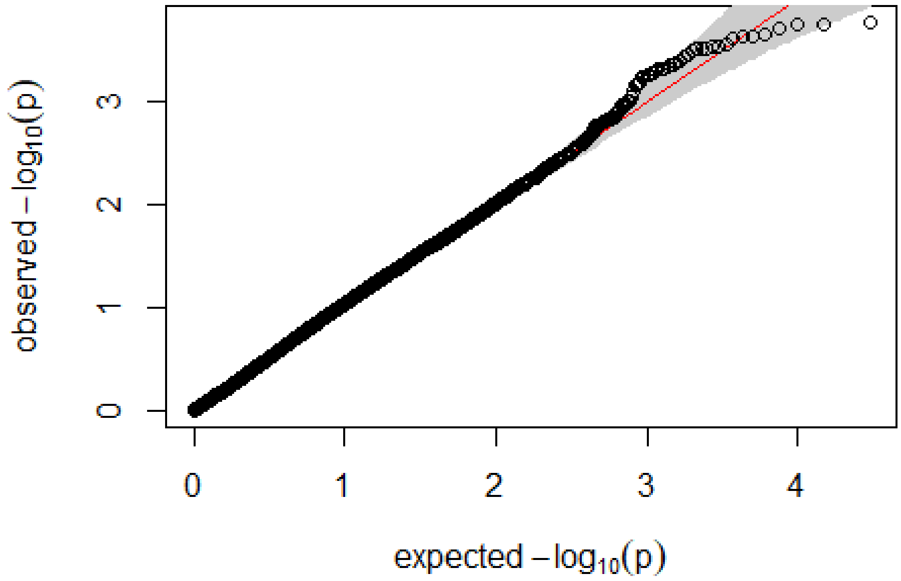

Manhattan and QQ plots for trait SPH
No SNP adjusted p-values exceed the FDR threshold of 0.05

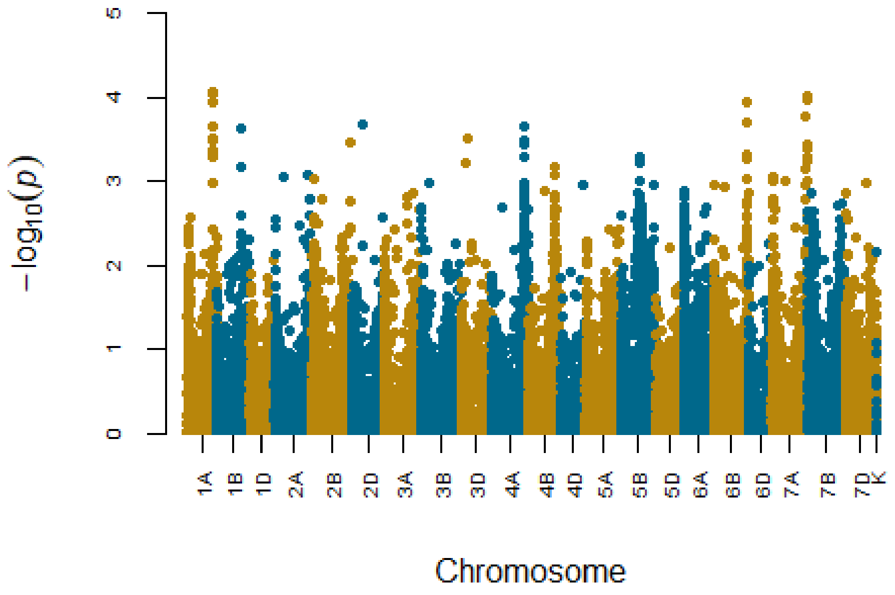

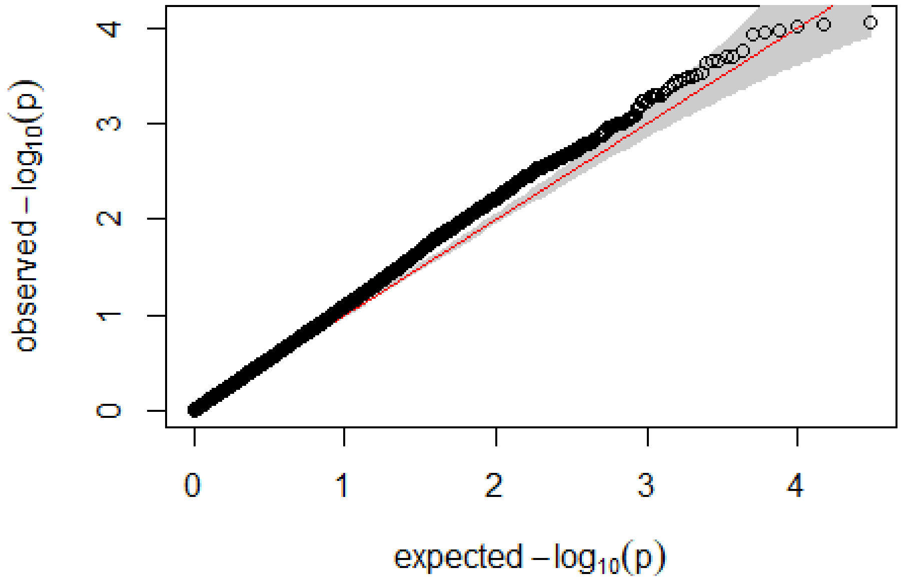

Manhattan and QQ plots for trait SSQM

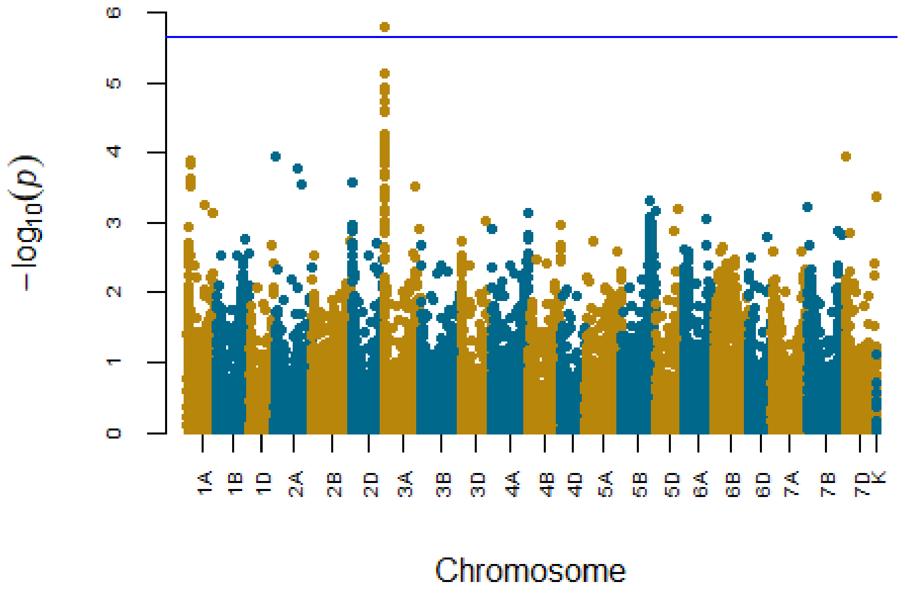

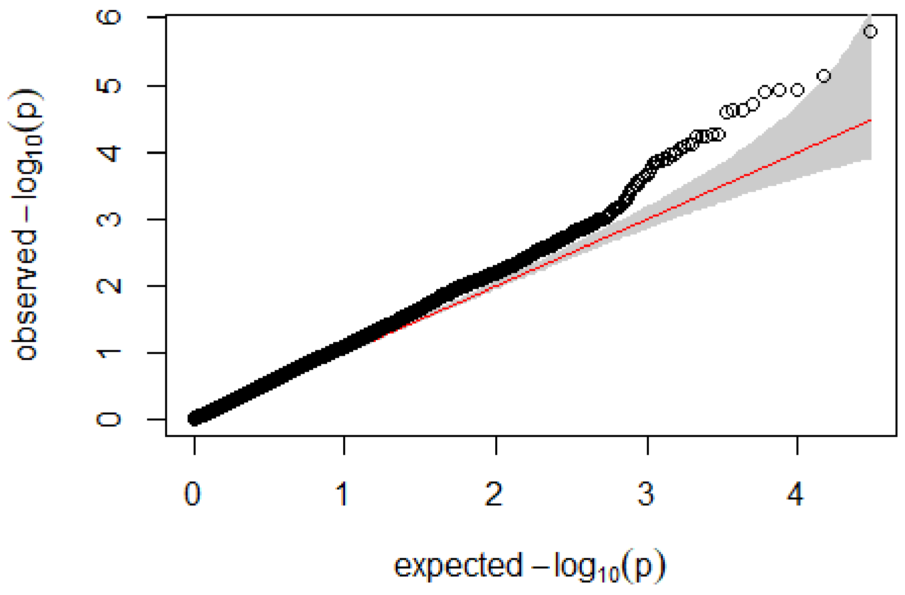

Manhattan and QQ plots for trait STARCH
No SNP adjusted p-values exceed the FDR threshold of 0.05

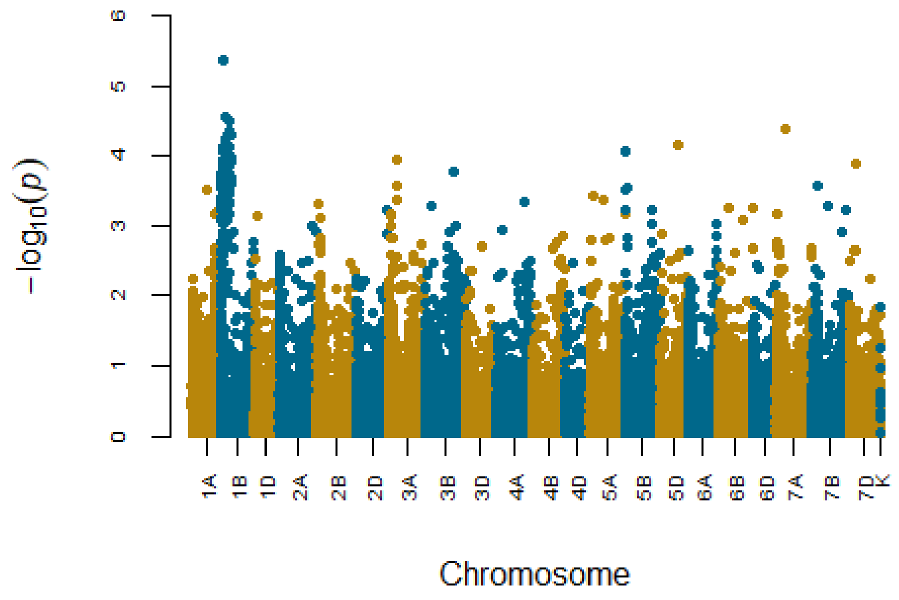

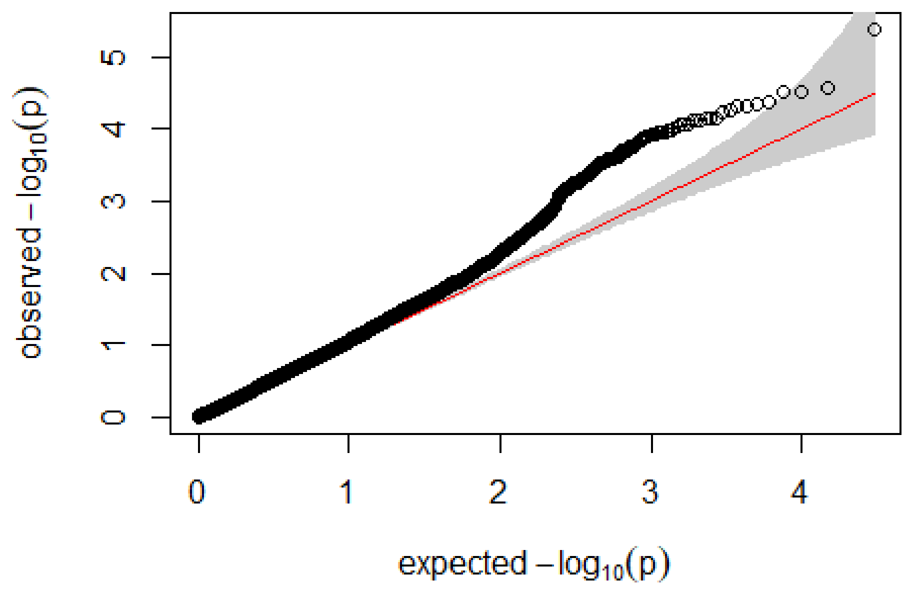

Manhattan and QQ plots for trait TKW

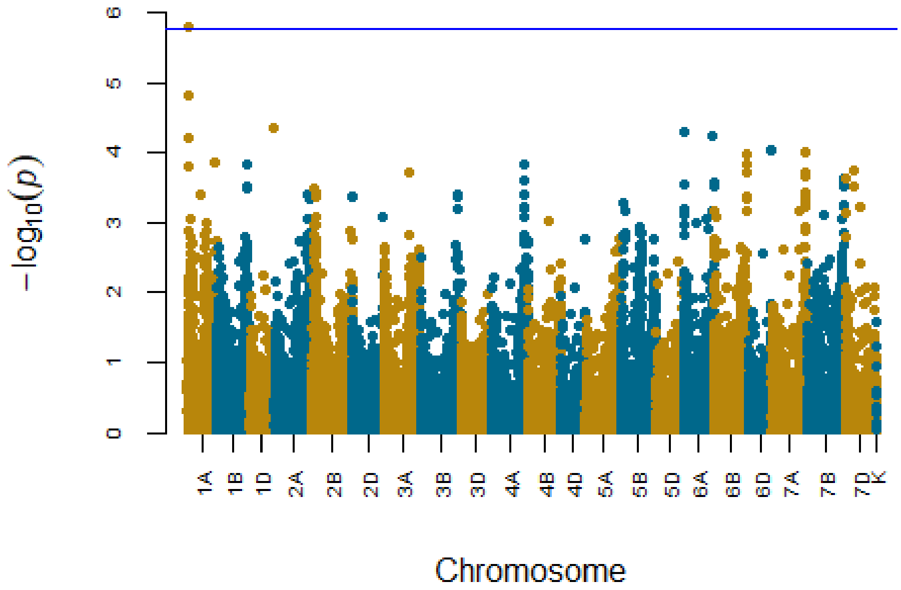

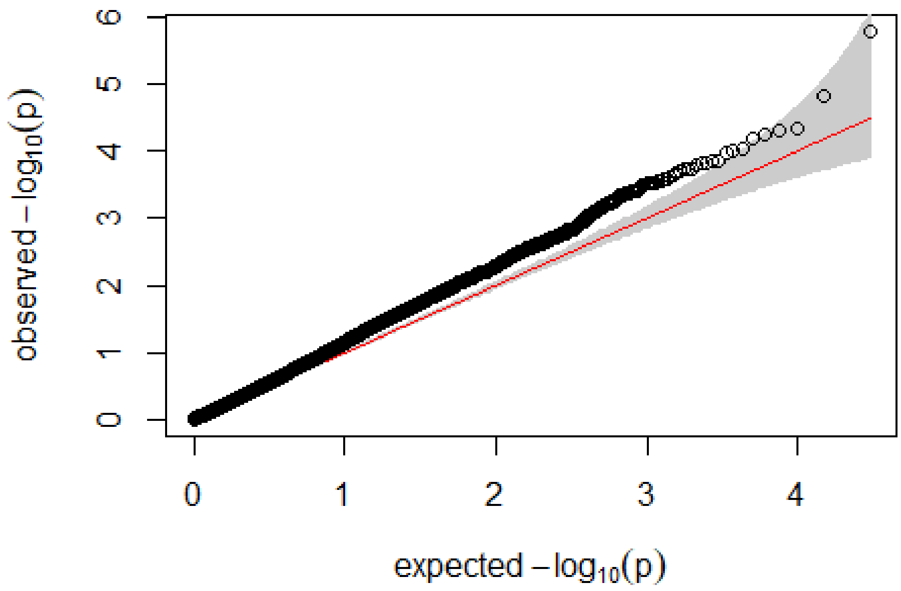

Manhattan and QQ plots for trait TWT

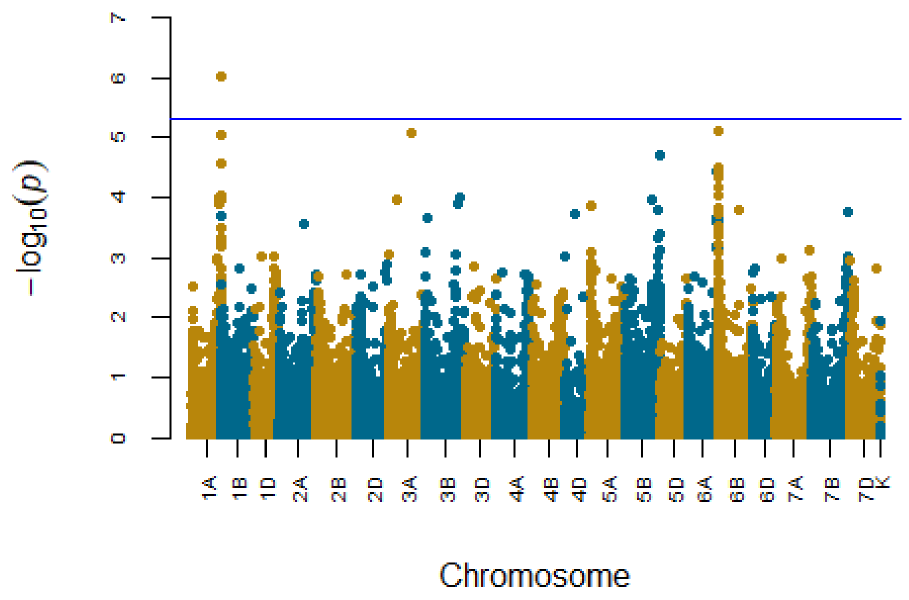

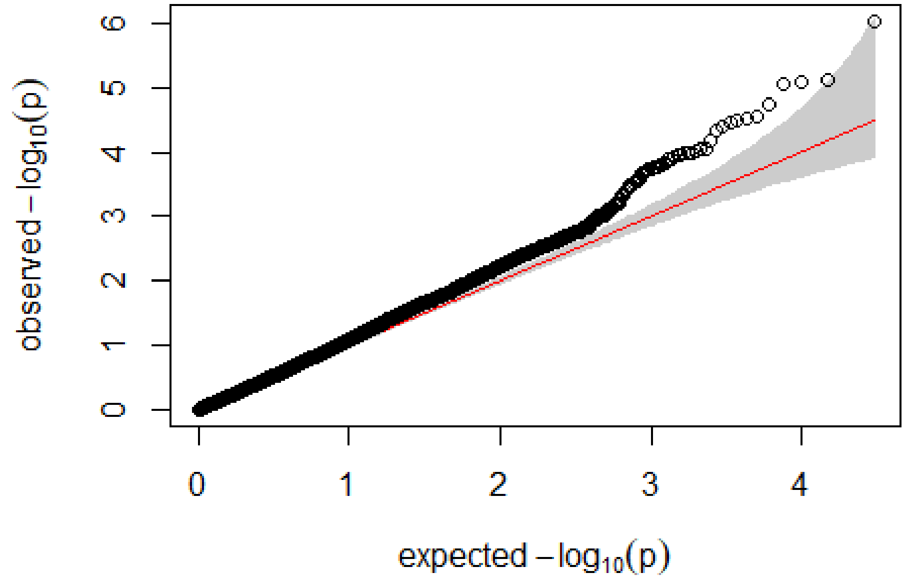

Manhattan and QQ plots for trait YLD

No SNP adjusted p-values exceed the FDR threshold of 0.05

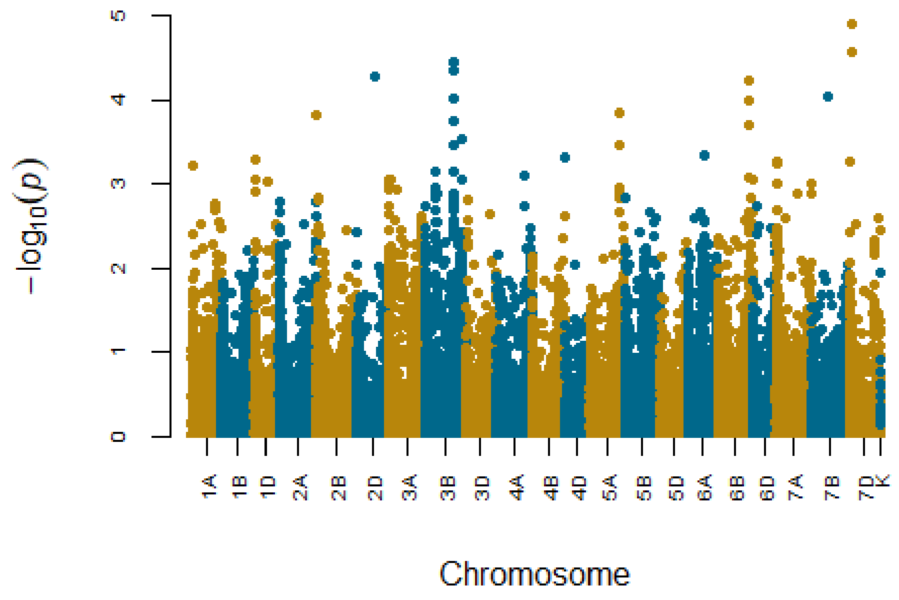

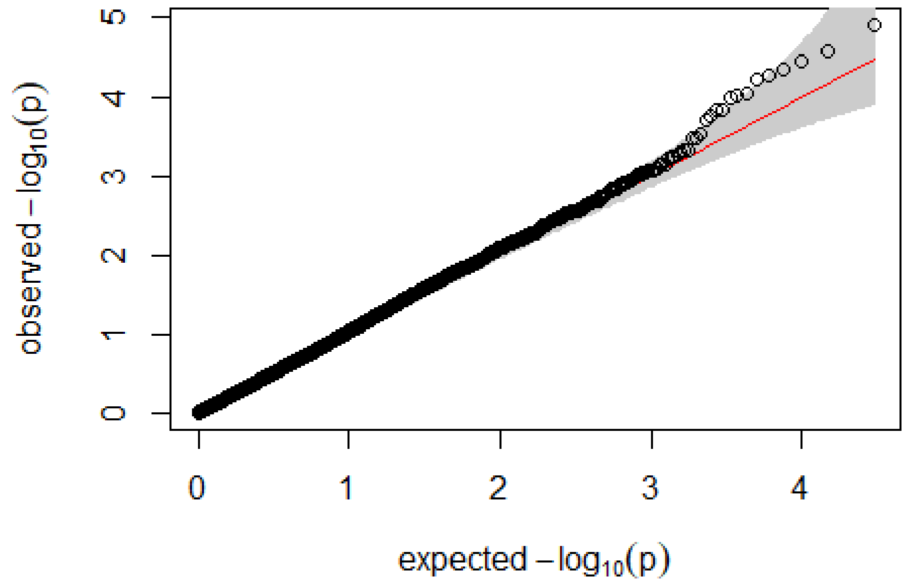

Supplement: S1 File — (PDF) [file pone.0208217.s008.pdf]
