# Supplementary material for: Genome-wide association studies for yield-related traits in soft red winter wheat grown in Virginia
Source: PLoS One. 2019 Feb 22;14(2):e0208217. doi: 10.1371/journal.pone.0208217 (PMC6386437; doi:10.1371/journal.pone.0208217)

Genome-wide association studies for yield-related traits in soft red winter wheat grown in Virginia

Brian P. Ward, Frederic L. Kolb, David A. Van Sanford, Gina Brown-Guedira, Priyanka Tyagi, Clay H. Sneller, and Carl A. Griffey

S2 File. Manhattan and QQ plots for SNP p-values generated by the FarmCPU algorithm.

Below are Manhattan and uniform distribution quantile-quantile (QQ) plots produced from the output of a GWAS analysis performed using the FarmCPU algorithm.

A total of 29,949 genotyping-by-sequencing (GBS) generated markers were included in the analysis.

A false-discovery rate (FDR) threshold was used to determine signficance for individual SNP p-values. A horizontal line is placed on the Manhattan plot only if at least one SNP's -log(p) value exceeds the FDR threshold.

Manhattan and QQ plots for trait FLSG

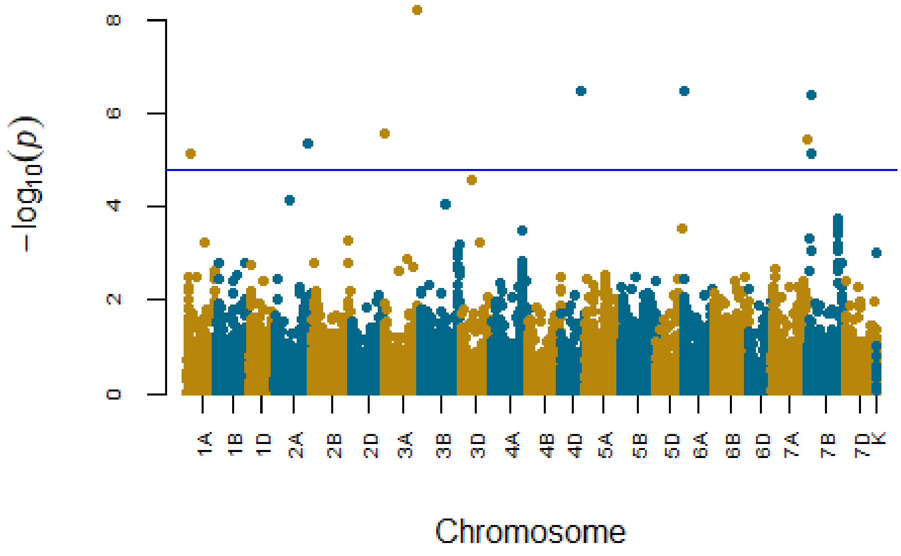

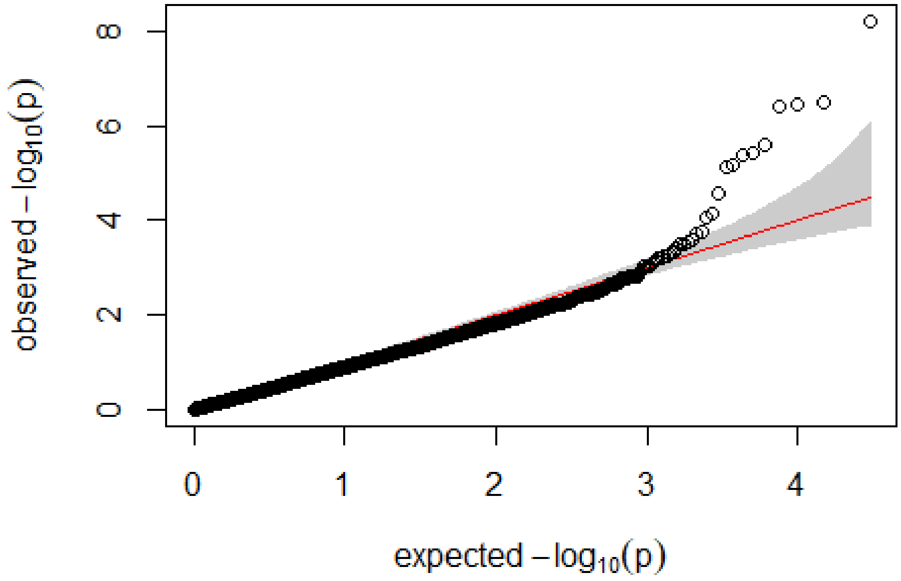

Manhattan and QQ plots for trait GSQM

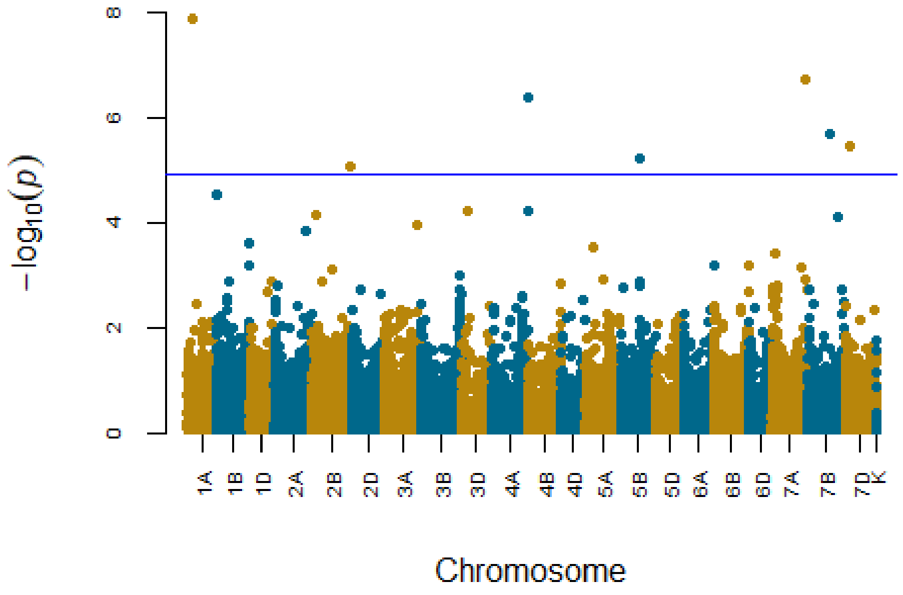

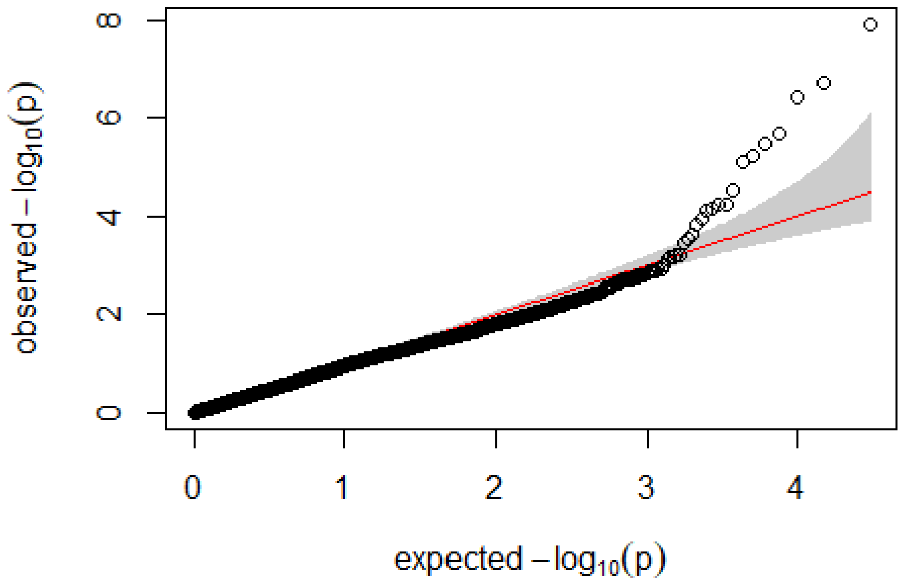

Manhattan and QQ plots for trait GW

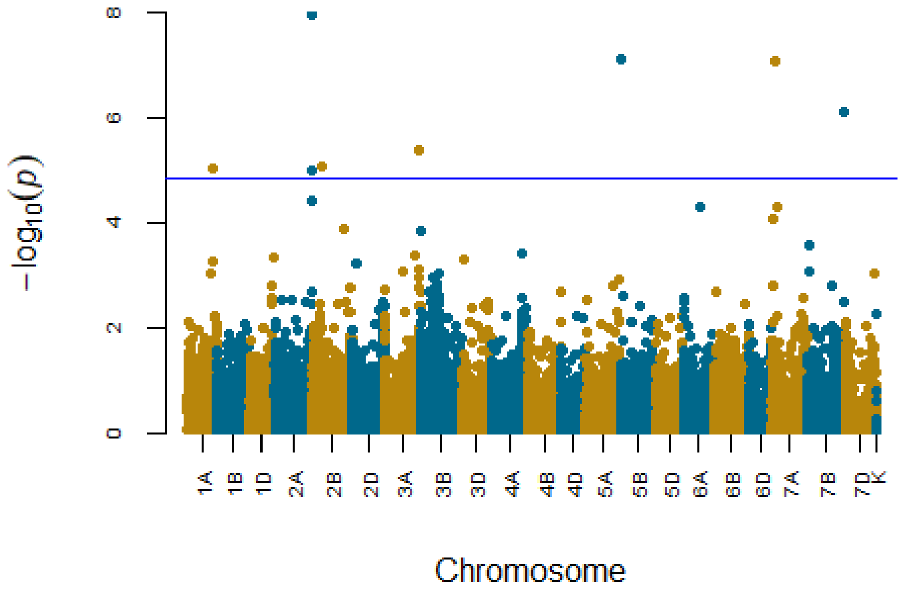

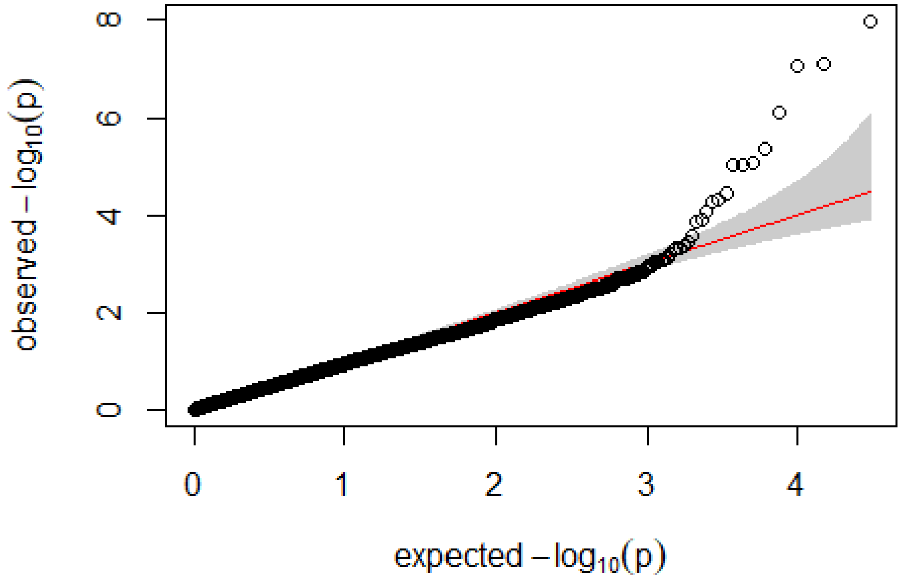

Manhattan and QQ plots for trait HD

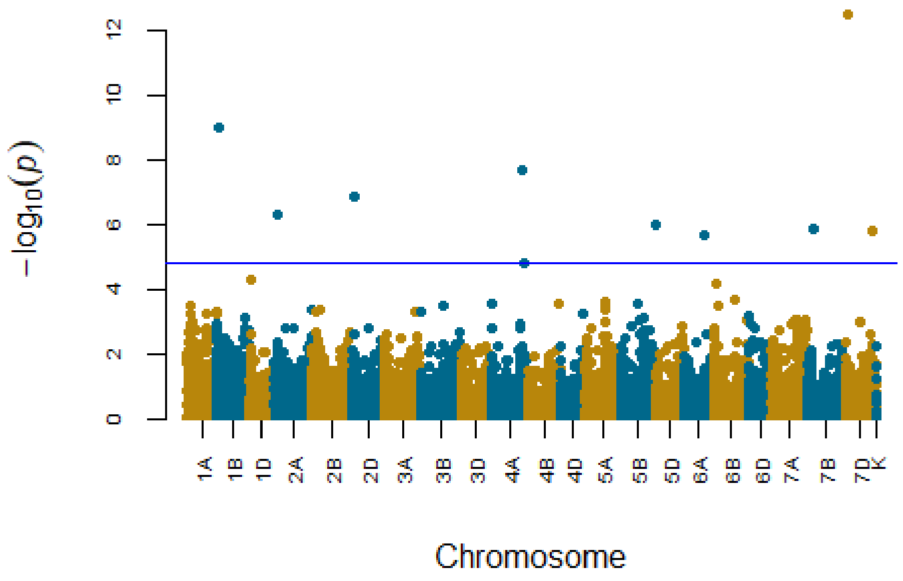

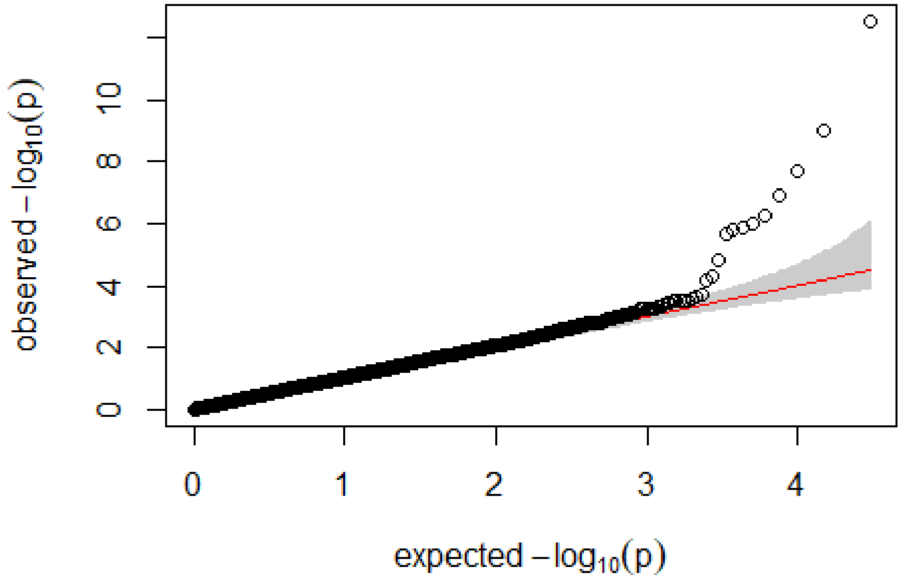

Manhattan and QQ plots for trait HT

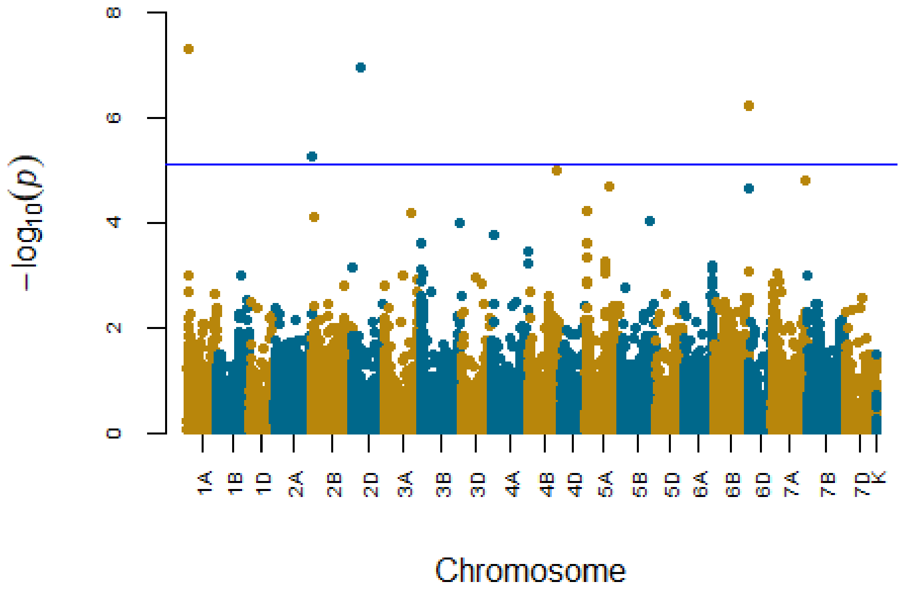

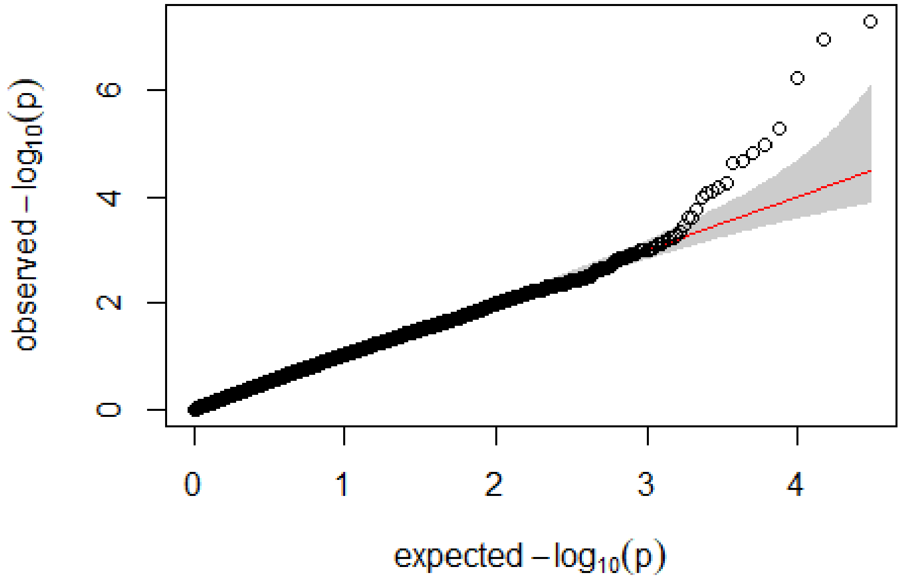

Manhattan and QQ plots for trait MAT

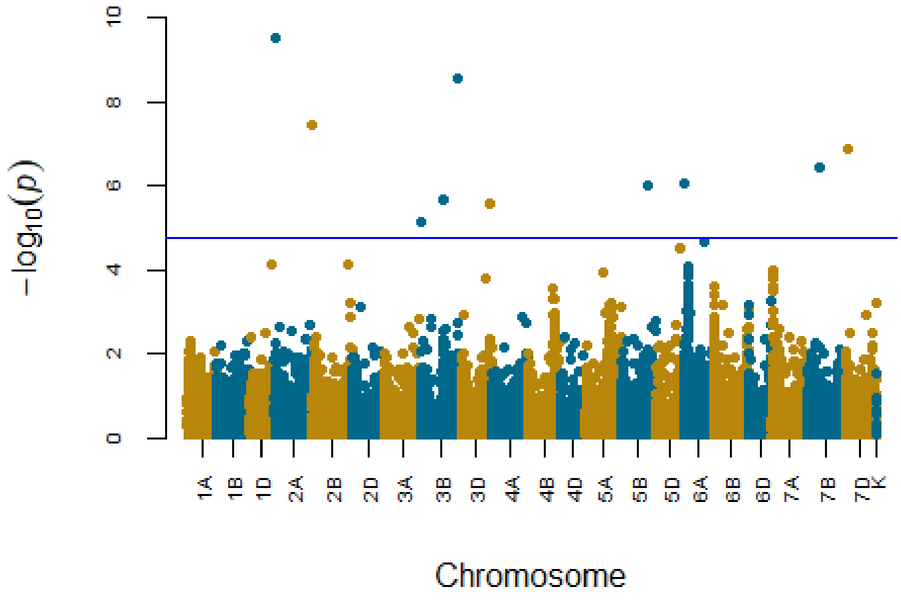

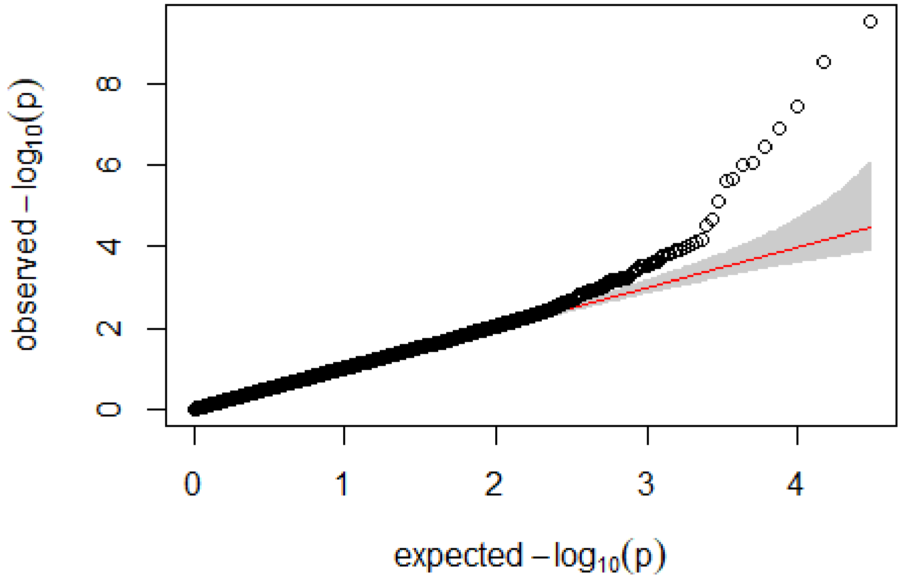

Manhattan and QQ plots for trait NDVI

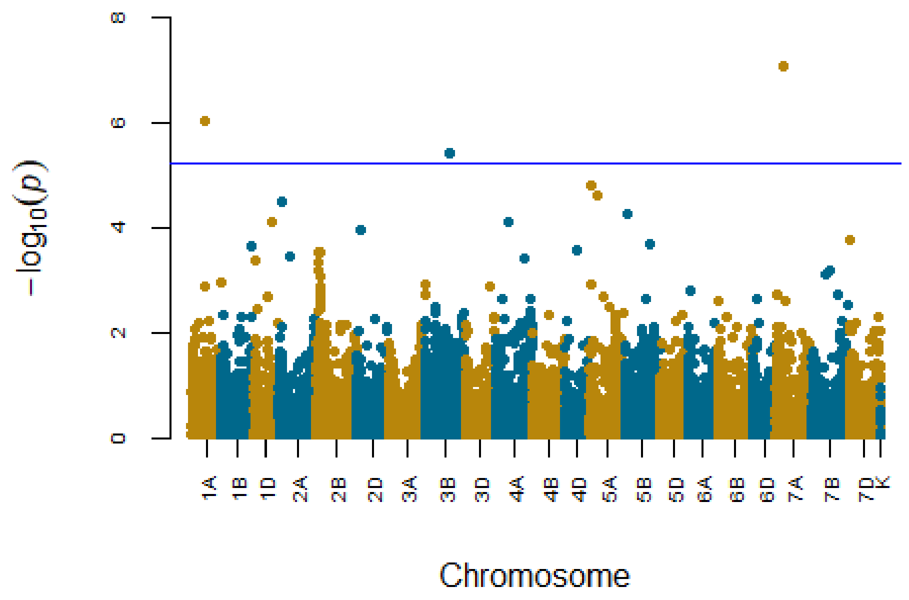

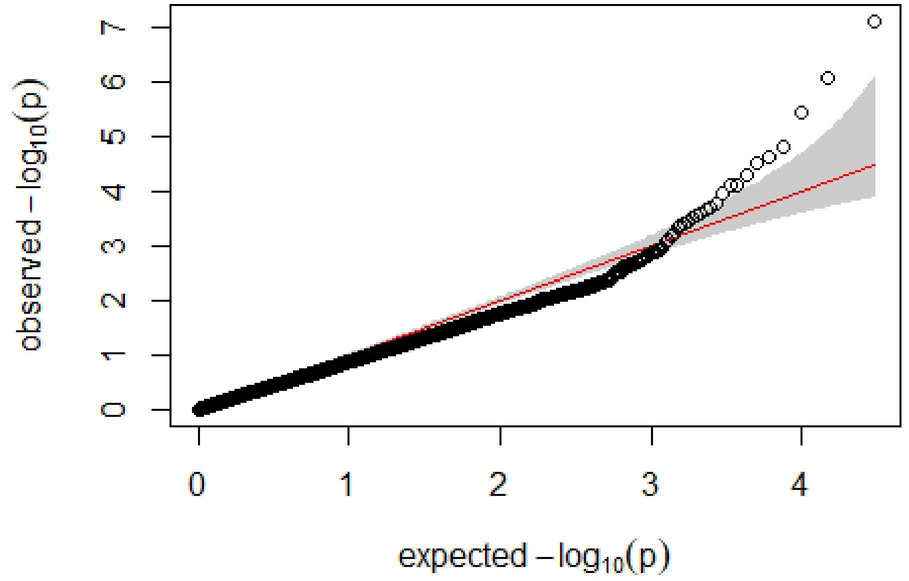

Manhattan and QQ plots for trait PROT

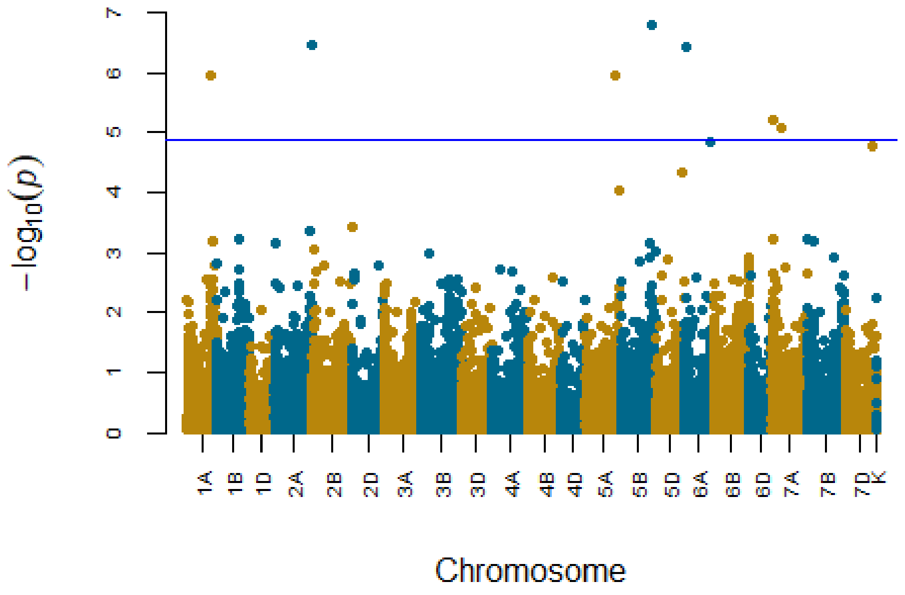

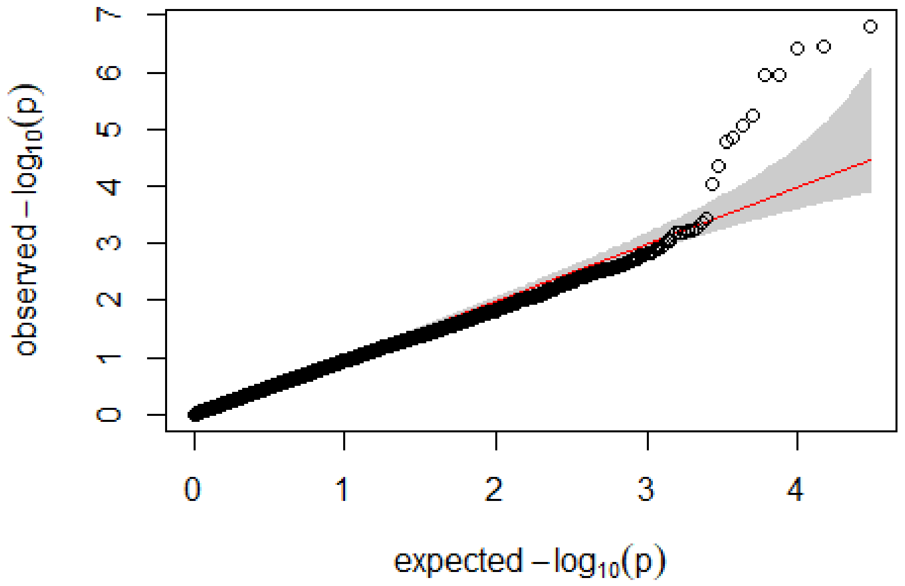

Manhattan and QQ plots for trait SPH

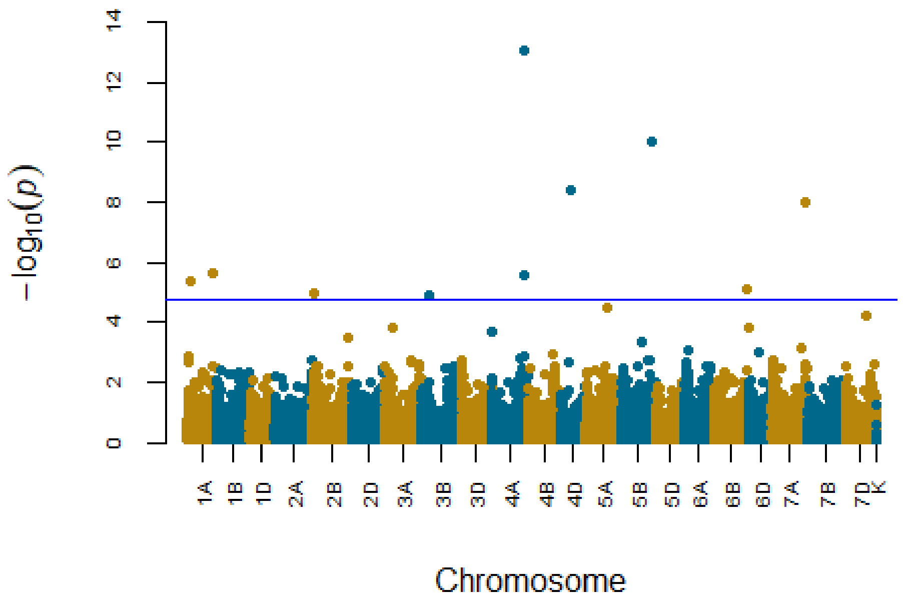

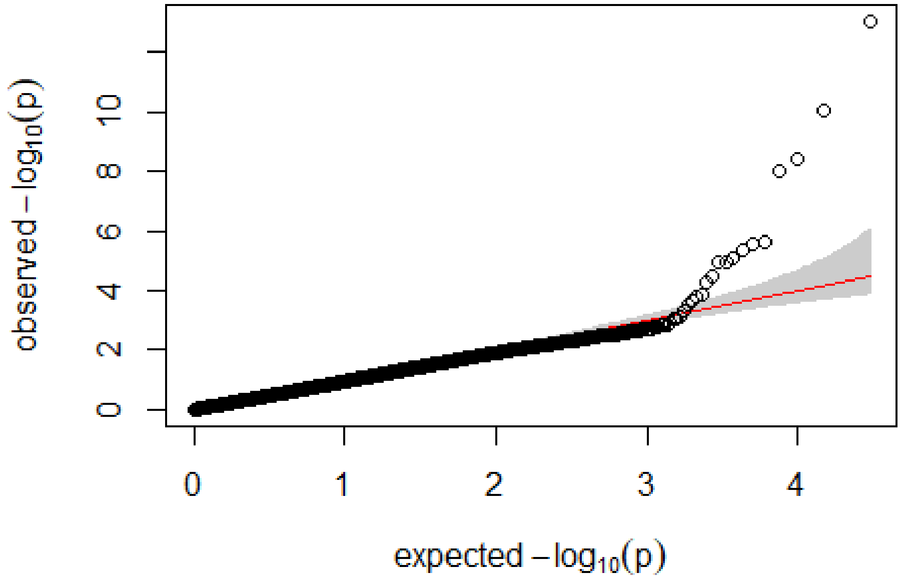

Manhattan and QQ plots for trait SSQM

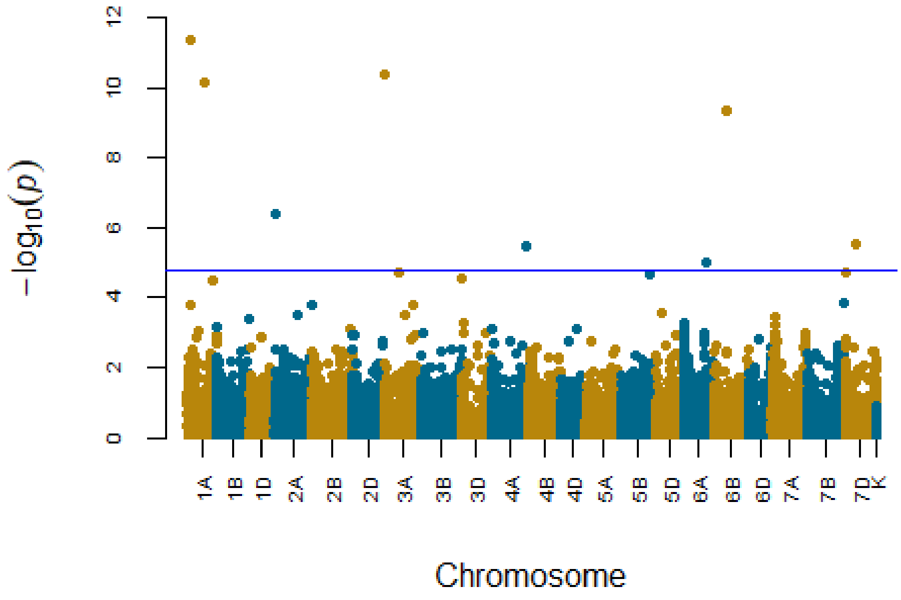

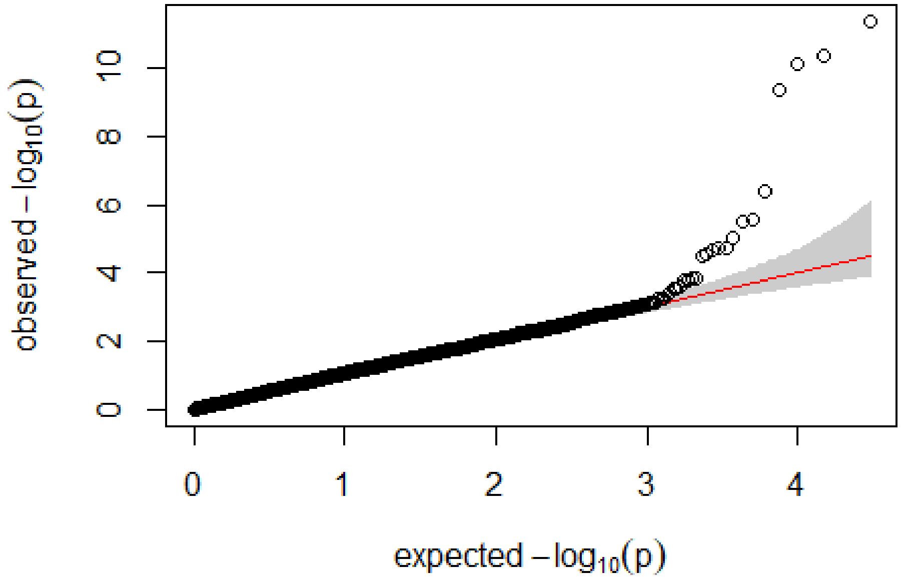

Manhattan and QQ plots for trait STARCH

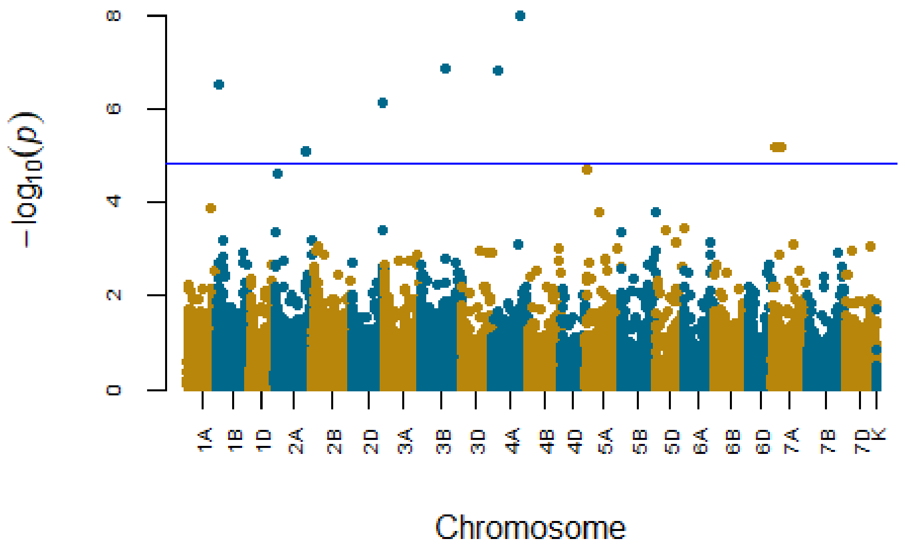

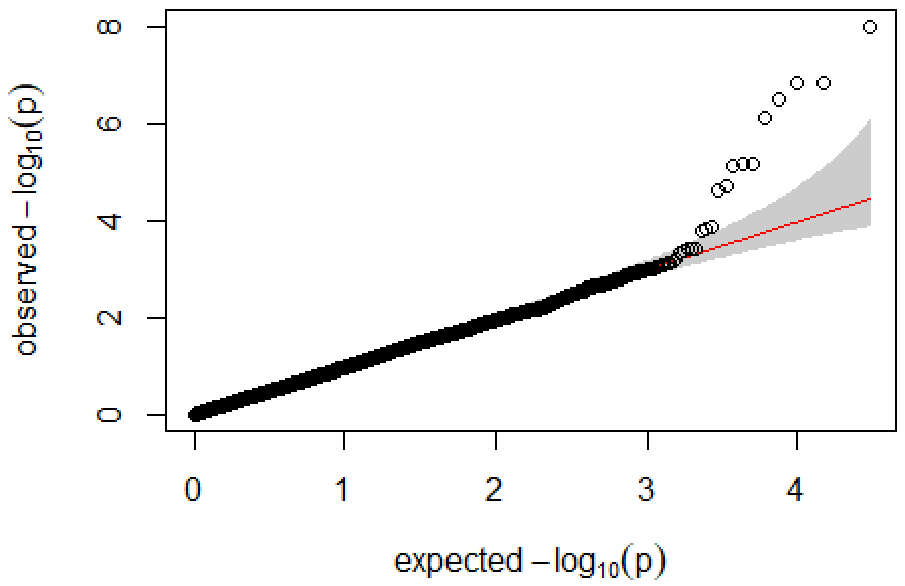

Manhattan and QQ plots for trait TKW

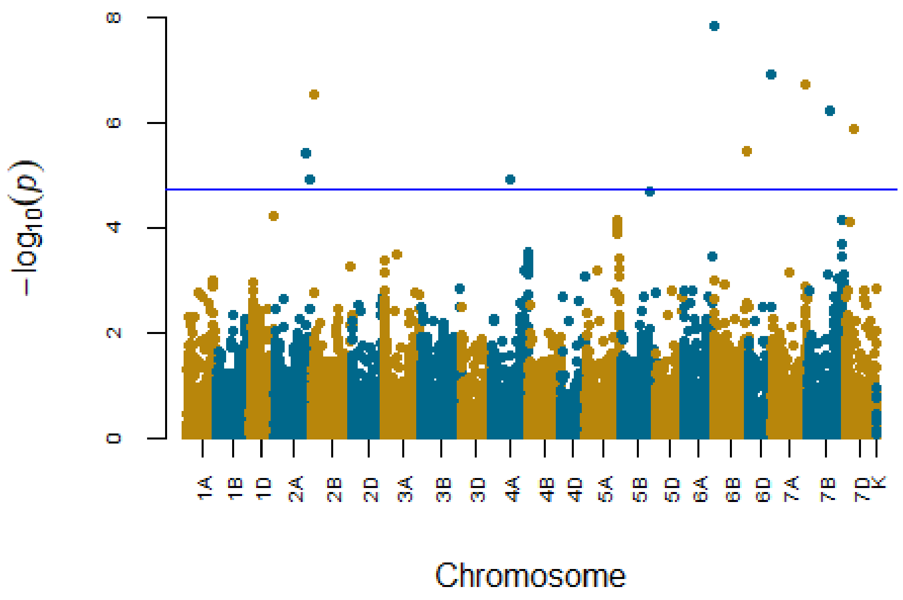

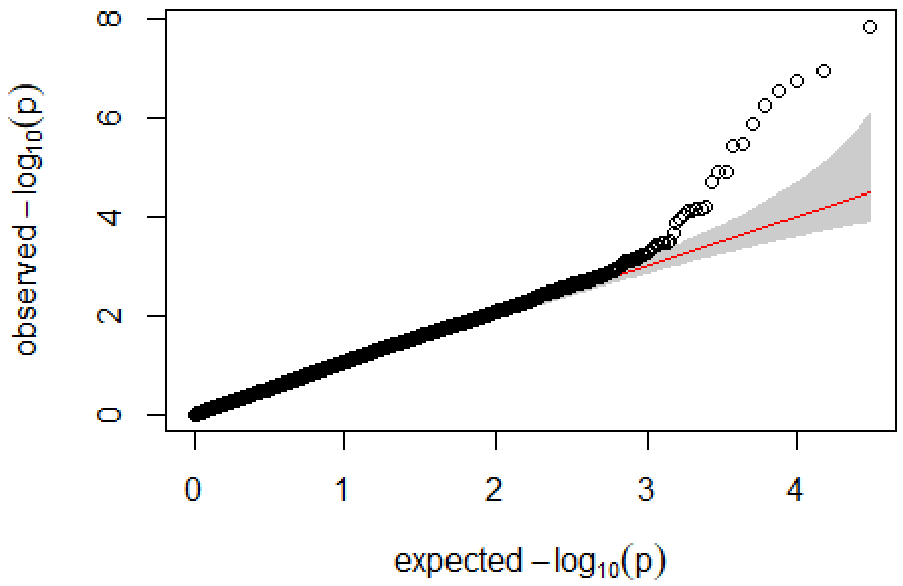

Manhattan and QQ plots for trait TWT

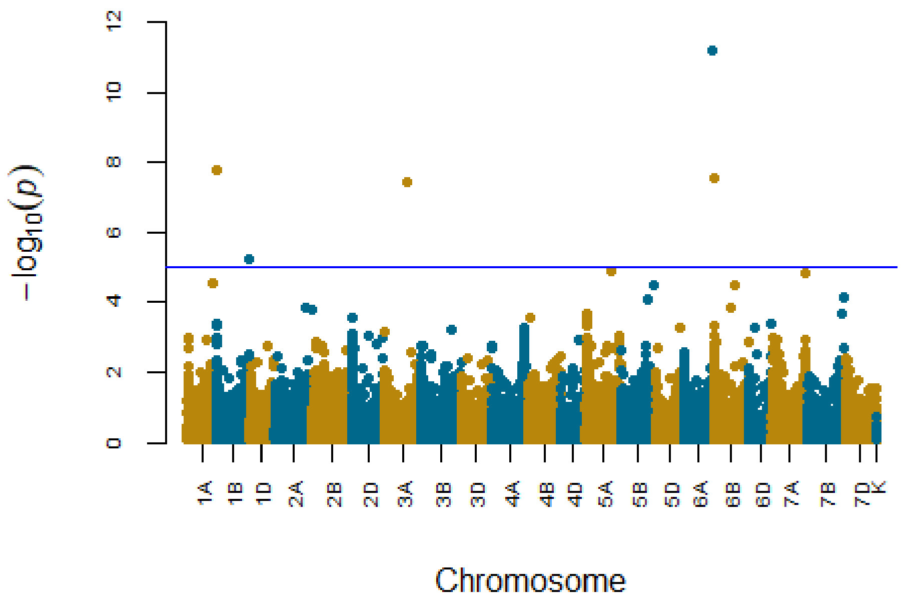

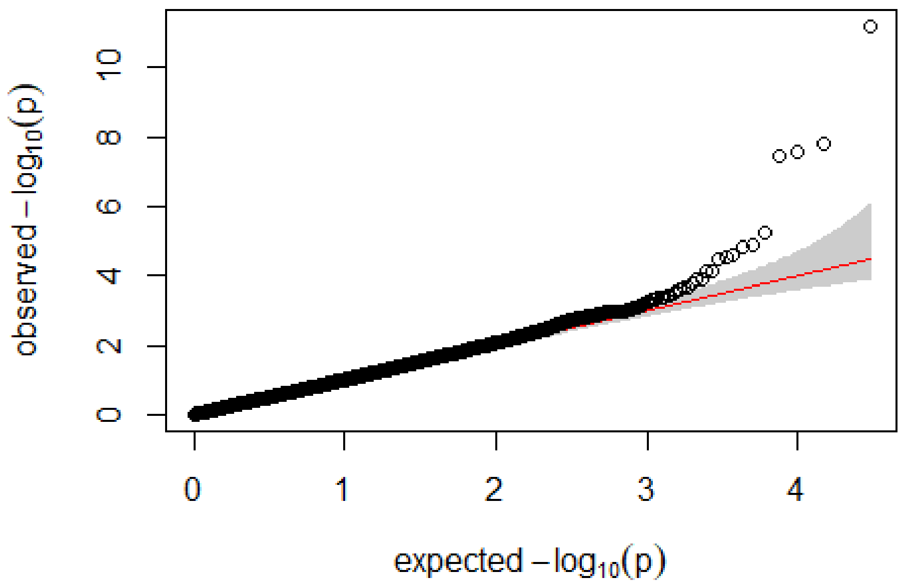

Manhattan and QQ plots for trait YLD

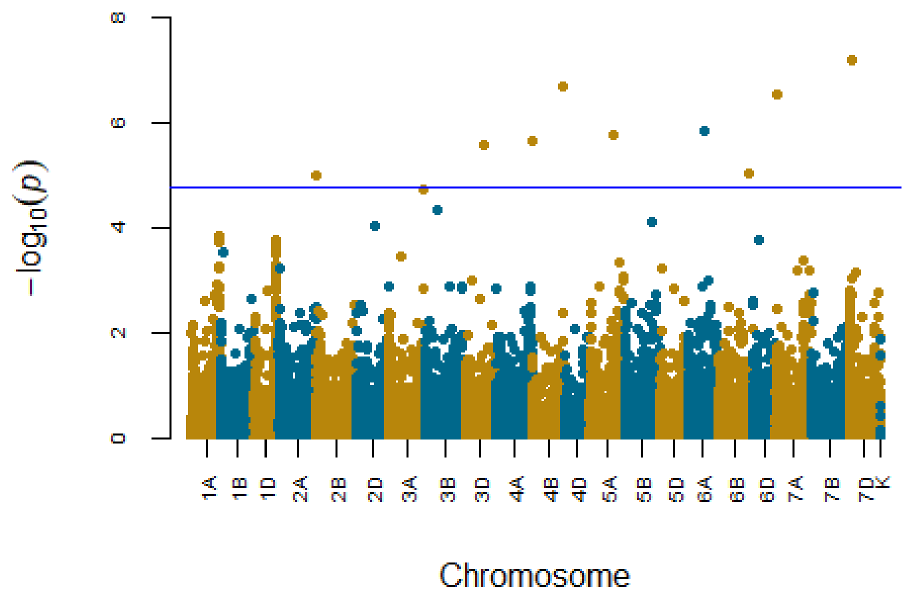

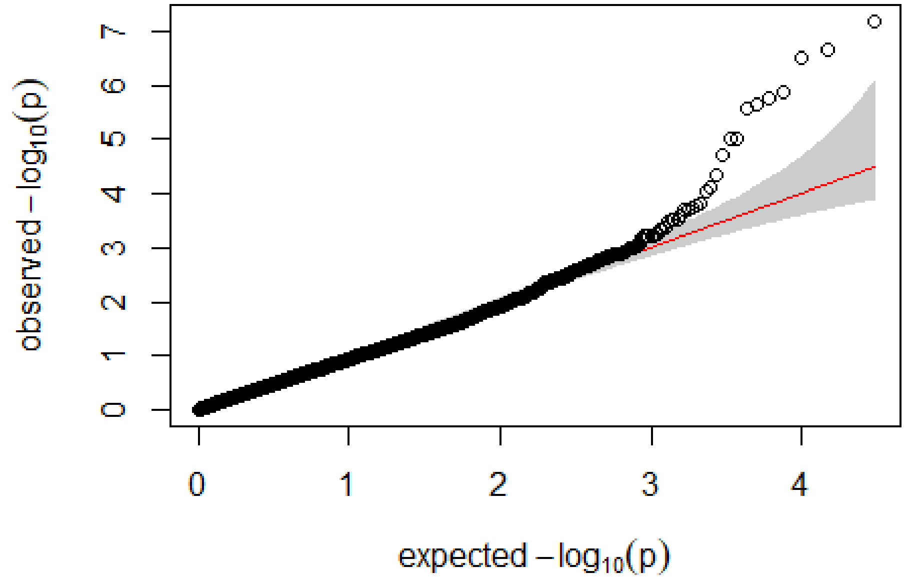

Supplement: S2 File — (PDF) [file pone.0208217.s009.pdf]
